# Next-generation sequencing analysis of circulating micro-RNA expression in response to parabolic flight as a spaceflight analogue

**DOI:** 10.1038/s41526-020-00121-9

**Published:** 2020-11-02

**Authors:** Peter Jirak, Bernhard Wernly, Michael Lichtenauer, Marcus Franz, Thorben Knost, Thaer Abusamrah, Malte Kelm, Nana-Yaw Bimpong-Buta, Christian Jung

**Affiliations:** 1 Clinic of Internal Medicine II, Department of Cardiology, Paracelsus Medical University of Salzburg, Salzburg, Austria; 2 Department of Internal Medicine I, Jena University Hospital, Friedrich Schiller University Jena, Jena, Germany; 3 Division of Cardiology, Pulmonology, and Vascular Medicine, Medical Faculty, University Duesseldorf, Duesseldorf, Germany

**Keywords:** Molecular medicine, Translational research

## Abstract

Understanding physiologic reactions to weightlessness is an indispensable requirement for safe human space missions. This study aims to analyse changes in the expression of circulating miRNAs following exposure to gravitational changes. Eight healthy volunteers (age: 24.5 years, male: 4, female: 4) were included. Each subject underwent 31 short-term phases of weightlessness and hypergravity induced by parabolic flight as a spaceflight analogue. At baseline, 1 and 24 h after parabolic flight, venous blood was withdrawn. Analysis of circulating miRNAs in serum was conducted by means of next generation sequencing. In total, 213 miRNAs were robustly detected (TPM > 5) by small RNA sequencing in all 24 samples. Four miRNAs evidenced a significant change in expression after adjusting for multiple testing. Only miR-223-3p showed a consistent significant decrease 24 h after parabolic flight compared to baseline values and values at 1 h after parabolic flight. miR-941 and miR-24-3p showed a significant decrease 24 h after parabolic flight compared to 1 h after parabolic flight but not to baseline values. miR-486-5p showed a significant increase 24 h after parabolic flight compared to 1 h after parabolic flight but not to baseline values. A target network analysis identified genes of the p53 signaling pathway and the cell cycle highly enriched among the targets of the four microRNAs. Our findings suggest cellular adaption to gravitational changes at the post-transcriptional level. Based on our results, we suggest a change in cell cycle regulation as potential explanation for adaptational changes observed in space missions.

## Introduction

Human space missions regained international focus in the last years. Besides an established Mars program by the National Aeronautics and Space Administration (NASA) and a manned mission to the moon planned by both the Chinese space agency and NASA, space flights are also entering the private sector. Therefore space medicine, representing an indispensable requirement for save and successful missions, gained major awareness^1,2^.

The adaptation of human organ systems following exposure to microgravity has been investigated in former studies. The most important changes comprise an increase in cardiac output and pulmonary flow as well as changes of the baroreceptor reflex and the cerebrovascular autoregulatory system^3–7^. Long term changes commonly affect bones and muscles atrophy. Additionally, an impairment of lung function and liver function as well as a dysregulation of the immune system have been described^8–11^. A decrease in hemoglobin and an increase in glomerular filtration rate (GFR) and levels of myoglobin were reported by our study group^12^. While adaptive responses have been described recently, the molecular background of these adaptations, necessary to understand the changes in physiology, remains topic to ongoing investigations. First studies showing an alteration of miRNA expression in T-cell activation as well as a change in miRNA signature potentially influencing TGF-Beta response, and fibroblast growth suggest a weightlessness induced change in microRNA (miRNA) expression patterns as a potential explanation^13–15^.

miRNAs are small, single stranded noncoding RNAs, usually between 19 and 24 nucleotides long^16^. They are found in both, plants and animals and probably represent a well-preserved system of gene regulation^17^. miRNAs originate from precursor molecules known as primary miRNAs (pri-miRNAs), which can span up to several hundred bases and undergo sequential processing via the ribonuclease III complexes “Drosha” and “Dicer” in the nucleus and cytoplasm, respectively^16,17^. After the processing, mature miRNAs bind to the RNA-induced silencing complex (RISC)^16,17^. Via a complementary sequence also known as the seed region (6–8 nucleotides long), the miRNA can guide the RISC to a target messenger RNA (mRNA), leading to translational repression^16,17^, a mechanism referred to as RNA interference^18^. Accordingly, miRNA expression levels represent a key factor in the regulation of gene expression on the posttranscriptional level, influencing cell function and differentiation as well as autoregulation of protein formation^18^. Of note, individual miRNAs can target a huge number of different genes and thus regulate the whole gene expression patterns instead of only one specific gene. Apart from their intracellular role, miRNAs are also present in a cell-free and circulating form^19^. While cell-free miRNAs can be found in different body fluids (e.g., saliva, spine fluid) circulating miRNAs are only found in the blood. Besides a passive leakage following apoptosis, necrosis, and inflammatory processes, the majority of circulating miRNAs depends on different carriers for active secretion to avoid degradation^19,20^. Such carriers comprise protein complexes, lipoproteins as well as extracellular vesicles such as exosomes and microvesicles^19,20^. However, the exact distribution across the carriers mentioned above and their potential influence on the mode of action of the respective miRNAs is still topic to investigation. Circulating miRNAs can be disseminated as autocrine, paracrine, and endocrine signaling molecules within the body^19,21^. Studies have shown that circulating miRNAs are fully functional at their recipient cells, with a similar effect on gene silencing as observed for cellular miRNAs^19,21^. Accordingly, circulating miRNAs represent an important mode of intercellular communication, with the possibility of donor cells being able to influence the gene expression and the microenvironment of distant recipient cells^16^. Several studies have shown that miRNA transcription is cell-type specific and highly dynamic, with transcriptional changes occurring within minutes following a stimulus^22^. However, while the mode of action of circulating miRNAs appears similar to that of cellular miRNAs, their secretion patterns can differ considerably. This fact must be considered when interpreting findings made for circulating miRNAs.

Considering the different physical requirements in microgravity, an involvement of miRNAs in weightlessness-induced changes is assumed and was already described in small studies^13–15^. However, further analysis on the influence of space travel on miRNA regulation is needed to better understand the involved adaptational processes on a post-transcriptional level. In the present study, we hypothesized that exposure to gravitational changes might lead to rapid changes in intracellular miRNA profiles in various cell types, which would alter miRNA release from cells. Accordingly, we aimed for an analysis of circulating miRNA expression following exposure to gravitational changes induced by parabolic flight as a spaceflight analog.

## Results

### Baseline characteristics

In total, eight study participants were included in the study, four male and four females. The median age was 24.5 years. Baseline characteristics are depicted in Tables 1 and 2.

**Table 1:** Baseline characteristics + laboratory parameters and miRNA levels at baseline/1 h after parabolic flight/24 h after parabolic flight.

Parameter	Timepoint	Median	IQR
Male/female sex *n* (%)	Baseline	4 (50)/4 (50)	
Age (years)	Baseline	24.5	12.5
Weight (kg)	Baseline	80.5	27
Height (cm)	Baseline	177.5	17
Creatinine (µmol/l)	Baseline	72.93	7.52
	1 h after parabolic flight	66.3	8.18
	24 h after parabolic flight	67.63	13.26
GFR (ml/min/1,73 m²)	Baseline	93.95	26.4
	1 h after parabolic flight	106.3	34.45
	24 h after parabolic flight	106.15	16.35
CRP (mg/l)	Baseline	2	1.75
	1 h after parabolic flight	1.5	1.75
	24 h after parabolic flight	1.5	1.75
BNP (pg/ml)	Baseline	9.5	2.75
	1 h after parabolic flight	10	3
	24 h after parabolic flight	9	9
CK (U/l)	Baseline	113	130.75
	1 h after parabolic flight	120	121.5
	24 h after parabolic flight	141	125.5
Myoglobin (µg/l)	Baseline	33.5	24.6
	1 h after parabolic flight	40.35	43.7
	24 h after parabolic flight	30.3	47.2
mir-486-3p (TPM)	Baseline	807.113.74	600.797.18
	1 h after parabolic flight	503.349.41	331.243.28
	24 h after parabolic flight	1.067.561.35	629.299.14
mir-24-3p (TPM)	Baseline	414.17	180.57
	1 h after parabolic flight	554.23	211.55
	24 h after parabolic flight	318.63	99.47
mir-223-3p (TPM)	Baseline	705.58	972.14
	1 h after parabolic flight	665.82	1082.92
	24 h after parabolic flight	182.72	170.26
mir-941 (TPM)	Baseline	953.54	447.29
	1 h after parabolic flight	884.13	702.23
	24 h after parabolic flight	546.08	350.6

*µmol/l* micromole/liter, *ml/min/1.73* *m*²: milliliter/minute/1.73 square meters, *mg/l* milligram/liter, *pg/ml* picogram/milliliter, *U/l* units/litre, *µg/l* microgram/liter, *TPM* tags per million.

**Table 2:** Baseline characteristics + baseline laboratory parameters of each individual study subject.

Study subject	sex (m/f)	age (years)	weight (kg)	height (cm)	Creatinine (µmol/l)	GFR (ml/min/1.73 m²)	CRP (mg/l)	BNP (pg/ml)	CK (U/l)	Myoglobin (µg/l)
P1	m	40	93	176	70.7	108	1	9	65	24.8
P3	m	22	86	188	78.7	107.8	1	9	96	38.7
P5	m	23	95	193	73.4	116	2	9	209	26.7
P7	f	23	54	164	65.4	98.3	1	12	91	24.8
P8	f	25	75	172	69.8	89.6	3	16	182	53.6
P12	f	24	64	174	72.5	86.5	2	11	130	38.3
P14	f	31	69	179	74.3	79.9	5.2	10	56	28.7
P15	m	37	90	190	98.1	75.3	2	9	304	86.7

*µmol/l* micromole/liter, *ml/min/1.73* *m*² milliliter/minute/1.73 square meters, *mg/l* milligram/liter, *pg/ml* picogram/milliliter, *U/l* units/litre, *µg/l* microgram/liter.

### Assessment of hemolysis

The degree of hemolysis in serum samples was determined using two different methods: spectrophotometric analysis of free hemoglobin (414 nm) and quantification of the level of miR-451a relative to the level of miR-23a (hemolysis ratio (ΔCq) = CqmiR-23a – CqmiR-451a), as reported by Blondal et al.^23^. In our studies both methods were applied. Supplement Fig. 1a presents levels for RNA spike-in and cDNA spike-in determined by RT-qPCR. The data indicate homogenous RNA extraction efficiency across all 24 samples and absence of enzyme inhibition. Next the hemolysis miRNA ratio was determined (Supplement Fig. 1b). None of the 24 samples showed a ratio above the report threshold of ΔCq > 7. Next, OD 414 was determined in all samples. Only one sample exceeded an absorbance of 0.3 but did not show a noticeable increase in the hemolysis miRNA ratio (Supplement Fig. 1c). As potential explanation, lipid contaminants but not hemolysis might have increased the OD414 in this sample. Finally, we compared the levels of miR-23a-3p, miR-451a, the hemolysis ratio and OD414 across the three groups (Supplement Fig. 1d). We did not observe a significant difference in the levels of these biomarker and indicators of hemolysis between the respective groups. Interestingly, a trend towards an increase in miR-23a-3p (not responsive to hemolysis) after 24 h was observed. Together, these results demonstrate that the quality of all 24 serum samples was not impaired by hemolysis and adequate for miRNA profiling by next-generation sequencing.

### Expression-analysis

Regarding miRNA analysis, 213 miRNAs were robustly (tags per million (TPM) > 5) detected in all eight subjects for each time point. RNA spike-in recovery was assessed by NGS to confirm homogenous RNA extraction efficiency (Supplement Fig. 2a). A summary of sequencing quality data and RNA classification tables are provided in Supplement Fig. 3a–g.

**Fig. 1:**
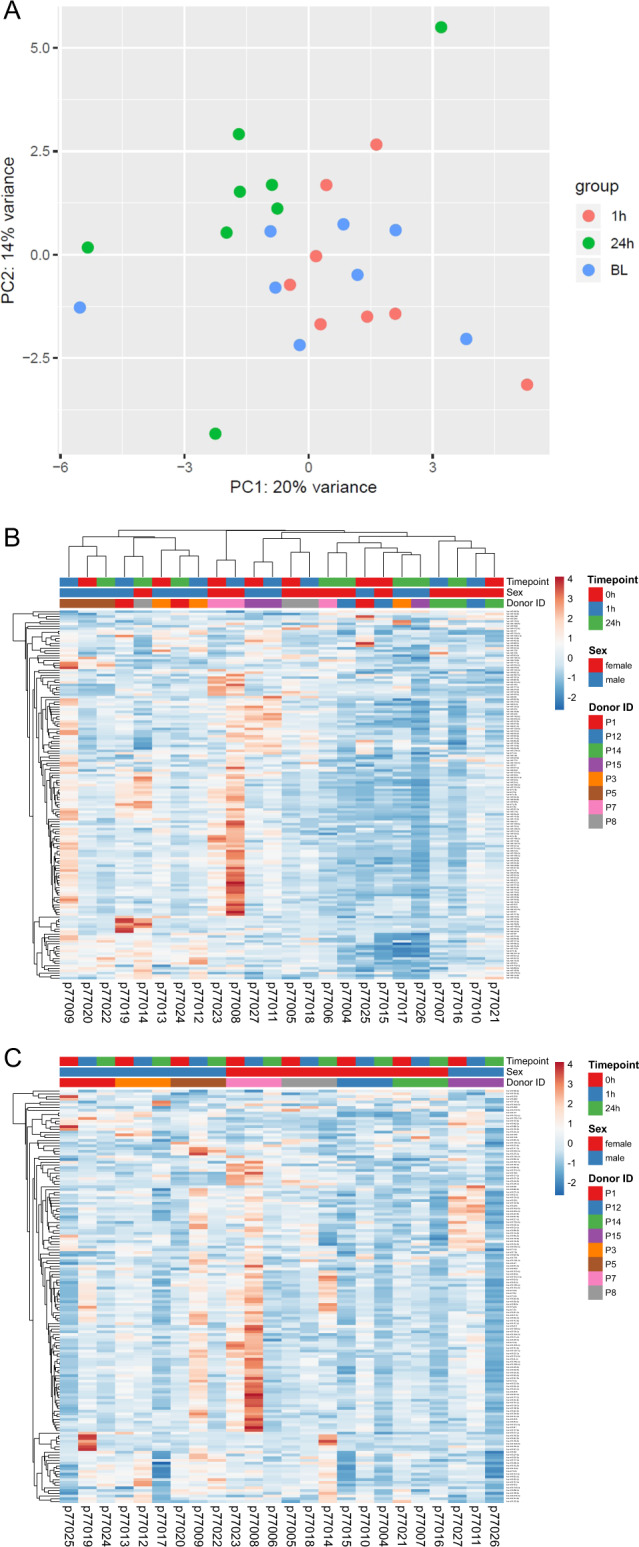
Unsupervised data analysis. **a** Principal component analysis (PCA) highlights microRNA patterns observed in subjects at baseline (BL), 1 and 24 h after parabolic flight. Data from 213 circulating microRNAs detected in all 24 samples by small RNAseq was used (regularized log tags per million data). The first and second principal component explain 20 and 14% of the variance, respectively. **b** Expression heatmap with unit variance scaling applied to rows (microRNAs). Both rows and columns are clustered using correlation distance and average linkage. Pearson correlation was used as correlation distance. **c** Expression heatmap with unit variance scaling applied to rows (microRNAs). Only rows are clustered using Pearson correlation as measure of distance and average linkage.

As depicted in Fig. 1a (principal component analysis, PCA), a change in the distribution of miRNA expression patterns for each respective timepoint was observed following exposure to gravitational changes. Besides, also donor-dependent differences between individuals were observed, as shown in the expression heatmap (Fig. 1b), where samples collected at baseline and 1 h cluster for several subjects (P3, P5, P7, P15, and P8). Figure 1c depicts the same heatmap ordered according to the donor number and time point to better illustrate donor-specific changes in miRNA patterns at 1 and 24 h compared to baseline.

**Fig. 2:**
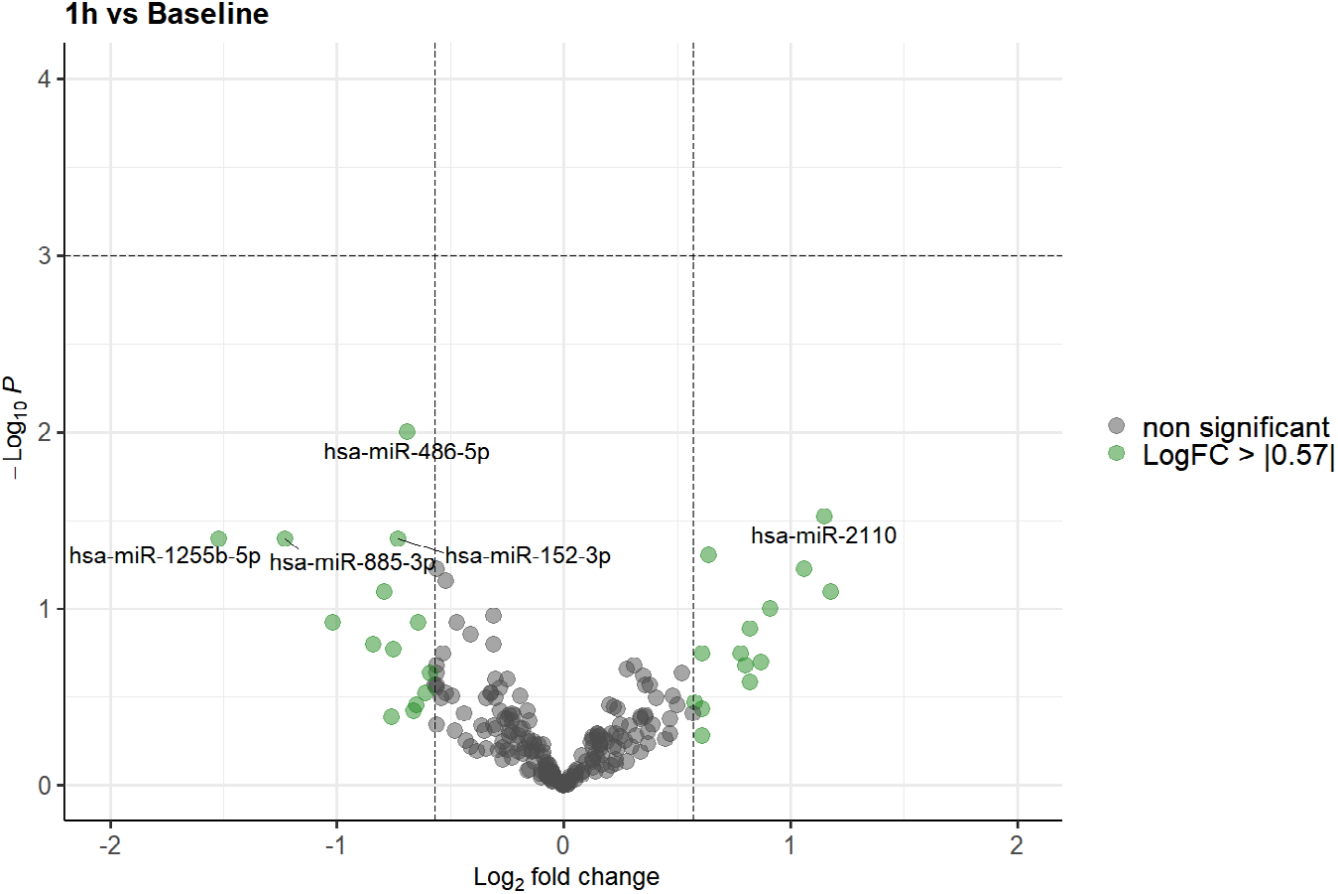
Volcano plot illustrating the effect of gravitational changes on serum levels of 213 miRNAs detected and quantified by next-generation sequencing. Plot depicts the relation between unadjusted *p*-value (*y*-axis, −log10 transformed) and effect size (*x*-axis, log_2_ transformed fold change) for serum microRNA of subjects at 1 h compared to baseline. MicroRNAs with a log_2_ fold change > 0.57 (1.5 fold linear change) are indicated in green. MicroRNAs with an adjusted *p*-value (FDR) of < 0.05 are highlighted in red. FDR false discovery rate.

**Fig. 3:**
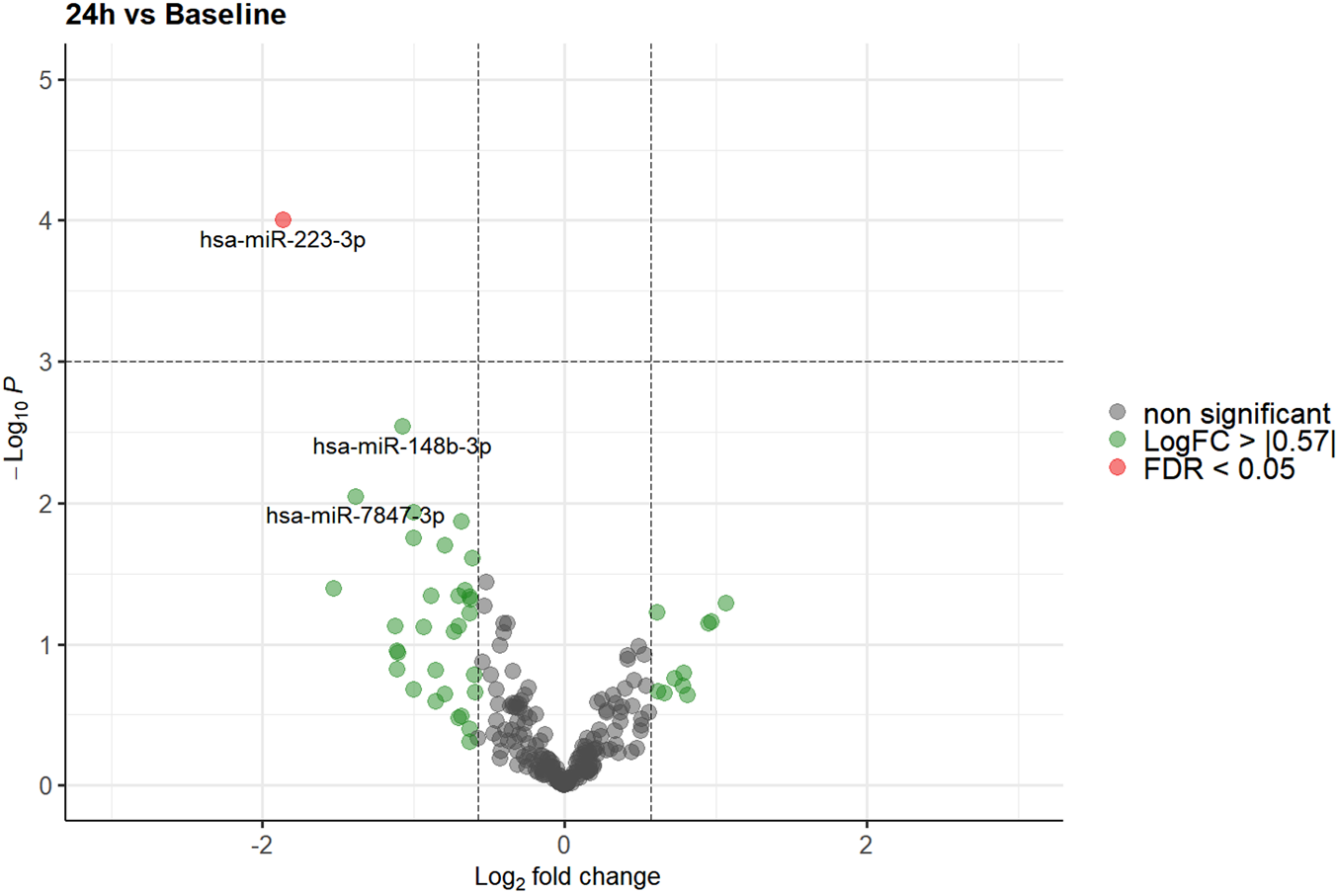
Volcano plot illustrating the effect of gravitational changes on serum levels of 213 miRNAs detected and quantified by next-generation sequencing. Plot depicts the relation between unadjusted *p*-value (*y*-axis, −log10 transformed) and effect size (*x*-axis, log_2_ transformed fold change) for serum microRNA of subjects at 24 h compared to baseline. MicroRNAs with a log_2_ fold change > 0.57 (1.5 fold linear change) are indicated in green. MicroRNAs with an adjusted *p*-value (FDR) of < 0.05 are highlighted in red. FDR false discovery rate.

**Fig. 4:**
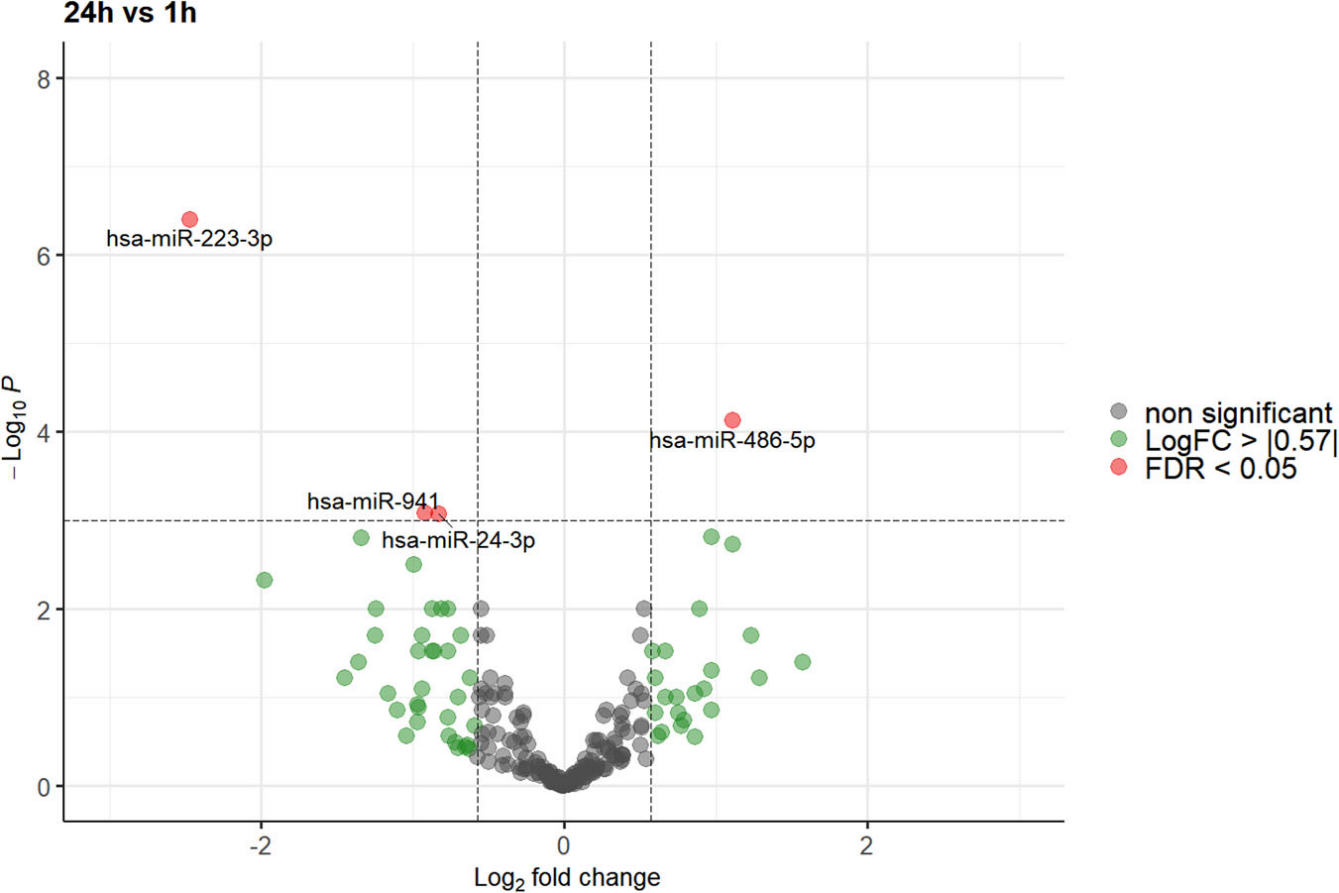
Volcano plot illustrating the effect of gravitational changes on serum levels of 213 miRNAs detected and quantified by next-generation sequencing. Plot depicts the relation between unadjusted *p*-value (*y*-axis, −log10 transformed) and effect size (*x*-axis, log_2_ transformed fold change) for serum microRNA of subjects at 24 h compared to 1h. MicroRNAs with a log_2_ fold change > 0.57 (1.5 fold linear change) are indicated in green. MicroRNAs with an adjusted *p*-value (FDR) of < 0.05 are highlighted in red. FDR false discovery rate.

Using the EdgeR GLM model, a significant alteration in miRNA expression (false discovery rate (FDR) < 0.05) was observed for 4 of the 213 miRNAs screened in our analysis 24 h after exposure to gravitational changes compared to baseline values and/or to levels 1 h after parabolic flight (miR-941, miR-24-3p, miR-486-5p, and miR-223-3p; Figs. 2 and 3). However, no significant differences in miRNA expression were observed between baseline values and levels 1 h after parabolic flight after adjusting for multiple testing (Fig. 4).

**Fig. 5:**
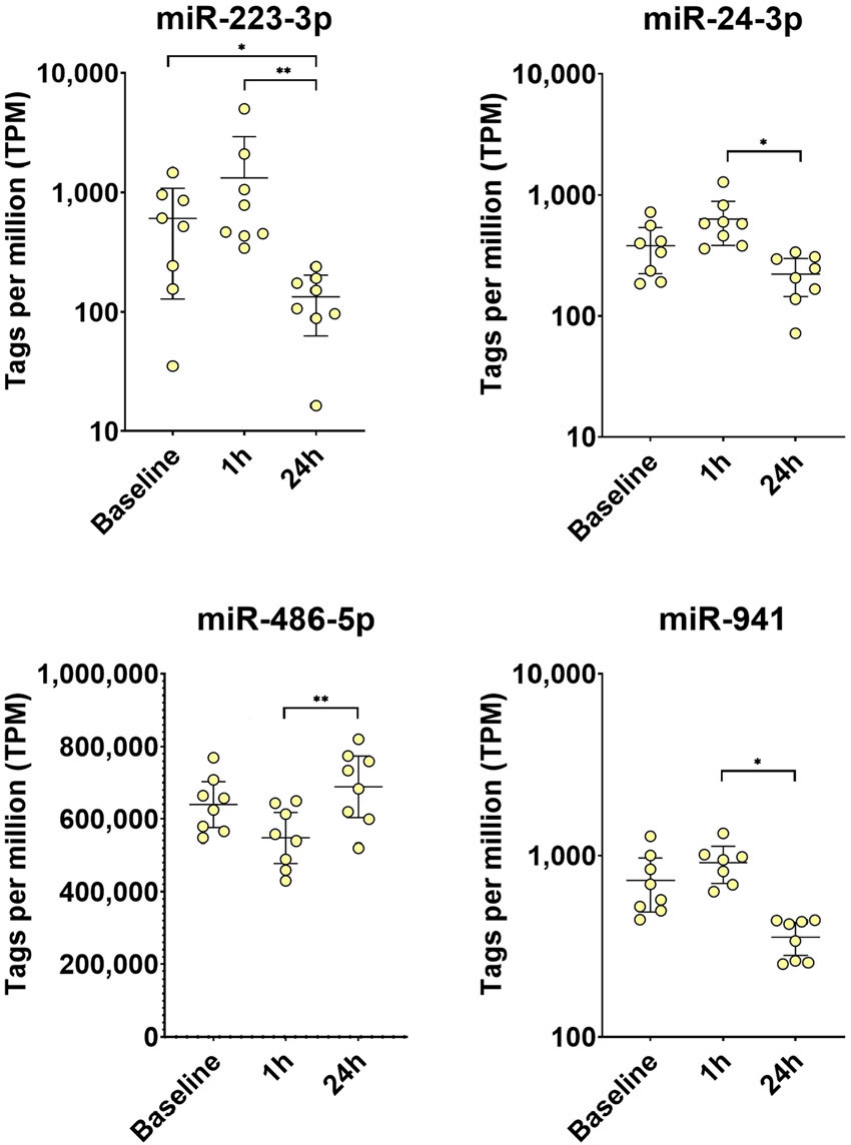
Levels of miRNAs with significant changes after 24 h compared to baseline or 1 h. EdgeR GLM method together with Benjamini–Hochberg adjustment for multiple testing. *adj-*p* value < 0.05, **adj-*p* value < 0.01.

As depicted in Fig. 5, after adjusting for multiple testing, miR-223-3p was the only miRNA to show a consistent significant change by means of a decrease 24 h after parabolic flight compared to levels at baseline and 1 h after parabolic flight. miR-24-3p and miR-941 showed a significant decrease at 24 h after parabolic flight compared to levels at 1 h after parabolic flight but not to baseline values. Inversely, miR-486-5p evidenced a significant increase at 24 h after parabolic flight compared to levels at 1 h after parabolic flight but again not to baseline levels. The detailed results of the EdgeR GLM method together with Benjamini–Hochberg adjustment for multiple testing are depicted in Table 3 and Fig. 5. A list of all miRNAs with TPM > 5 that were identified at each time point for each sample is depicted in Supplement Table 1.

**Table 3:** miRNA expression analysis.

	Abundance	1 h vs. Baseline			24 h vs. Baseline			24 h vs. 1 h		
microRNA ID	Avg TPM	logFC	*p* value	FDR	logFC	*p* value	FDR	logFC	*p* value	FDR
hsa-miR-223-3p	563	0,61	0.18	0.98	−1.86	<0.01	0.02	−2.47	<0.01	<0.01
hsa-miR-24-3p	364	0.31	0.21	0.98	−0.52	0.04	0.62	−0.83	<0.01	0.04
hsa-miR-486-5p	698.389	−0.69	0.01	0.98	0.42	0.13	0.78	1.11	<0.01	0.01
hsa-miR-941	686	0.24	0.37	0.98	−0.68	0.01	0.58	−0.92	<0.01	0.04

*Avg TPM* average tags per million, *logFC* log fold change, *FDR* false discovery rate.

**Fig. 6:**
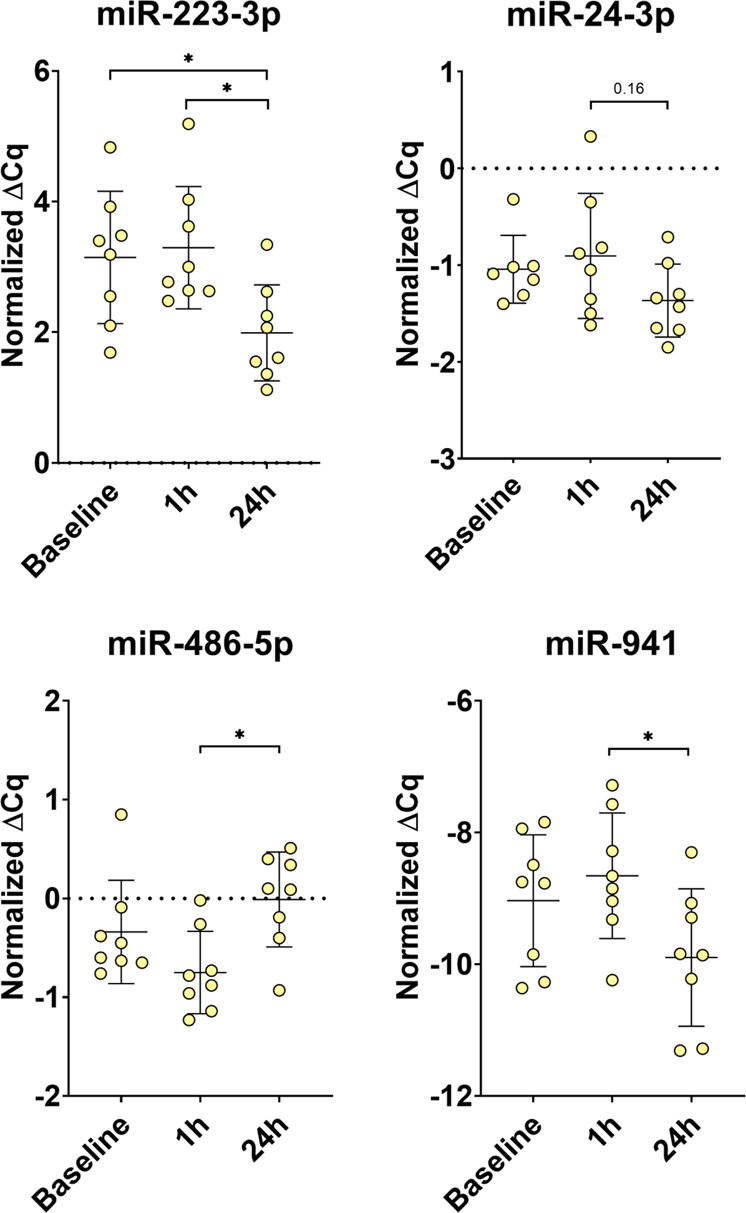
Technical verification of NGS results by RT-qPCR. Three miRNAs were selected for RT-qPCR analysis. Normalized delta Cq-values (ΔCq) values to the RNA spike-in as internal standard are shown. *adj-*p* value < 0.05, **adj-*p* value < 0.01.

In order to provide further evidence for the robustness of the NGS data, we have repeated the analysis of four miRNAs with significant (FDR < 0.05) change by RT-qPCR (Fig. 6). Again, miR223-3p was the only miRNA to show a consistent significant change with a decrease at 24 h after parabolic flight compared to levels at baseline and 1 h after parabolic flight. miR-941 evidenced a significant decrease at 24 h after parabolic flight compared to levels at 1 h after parabolic flight but not to baseline values. miR-486-5p evidenced a significant increase 24 h after parabolic flight compared to levels at 1 h after parabolic flight but not to baseline levels. In contrast, no significant change but only a trend towards a decrease was observed for miR-24-3p at 24 h after parabolic flight compared to levels at 1 h after parabolic flight.

### Correlation analysis

In a correlation analysis on baseline characteristics, we found no relevant correlations between miRNA expression and gender, age, or body mass index (BMI). Additionally, no relevant correlations were observed regarding miRNA expression and laboratory parameters. The detailed correlation analysis is given in the supplement Table 2 (miR-486-5p), 3 (miR-24-3p), 4 (miR-223-3p), and 5 (miR-941).

### Target network analysis

**Fig. 7:**
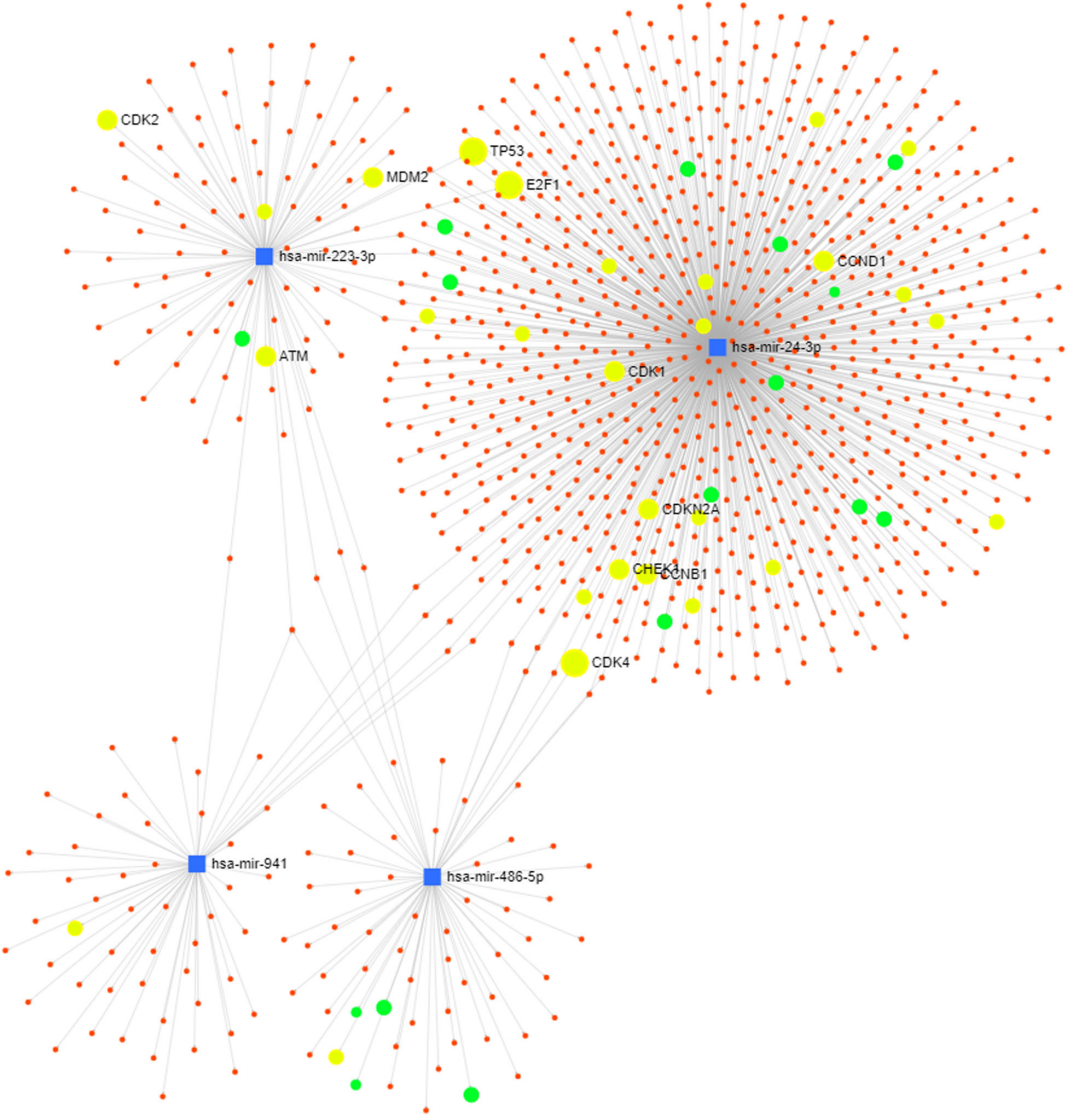
Target network analysis. Target network analysis for the four differentially regulated miRNAs—p53 signaling pathway (blue), cell cycle (green).

We performed a target network analysis for the four differentially regulated miRNAs using miRNet. We identified that genes from two KEGG pathways, the p53 signaling pathway (*p* < 0.0001) and the cell cycle (*p* < 0.0001) were highly enriched (hypergeometric test and empirical sampling) among the targets of the four microRNAs (Fig. 7 and Supplement table 6). We observed that TP53 and E2F1 (miR-223-3p and miR-24-3p), and CDK4 (miR-24-3p and miR-486-5p) were targeted by two of the four miRNAs. All other genes were only targeted by one miRNA. miR-24-3p was found to be the most important regulator of both pathways. A detailed list of genes regulated by at least one of the four miRNAs is given in the Supplement Tables 7a (p53) and 7b (cell cycle).

## Discussion

In this study, we present an analysis of the expression profile of circulating miRNAs following exposure to gravitational changes induced by parabolic flight as a space-flight analog. Exposure to weightlessness induces adaptations in autoregulatory mechanisms and functions of virtually all organs, with the cardiovascular system being responsible for most severe complications^3,5,24^. Adaptations to microgravity have been reported in previous studies especially in the cardiovascular field. First studies showing an alteration of miRNA expression in T-cell activation as well as a change in miRNA signature potentially influencing TGF-Beta response and fibroblast growth suggest a weightlessness induced change in micro-RNA expression patterns as a potential explanation^13,14^.

In contrast to recent research targeting miRNA expression following spaceflight and exposure to gravitational changes, our study aimed for the analysis of circulating miRNAs from serum. Accordingly, our results reflect the secretion of circulating miRNAs following exposure to gravitational changes but limited conclusion can be drawn with regards to definite intracellular processes^19^. Thus, also direct comparability of our results with former projects is limited^16,19,21^.

In our study, four miRNAs, miR-941, miR-24-3p, miR-486-5p, and miR-223-3p evidenced a significant change following exposure to gravitational changes after adjusting for multiple testing. Of note, only miR-223-3p showed a consistent significant decrease 24 h after parabolic flight compared to both baseline levels and levels 1 h after parabolic flight. For the rest of the miRNAs, significant changes were only observed between 1 and 24 h. Accordingly, the effect of these changes is assumed to be lower compared to miR-223-3p. However, the results of the RT-qPCR analyses in our study point emphasize the consistency of our results. Of note, hemolysis was ruled out as a potential confounder by spectrophotometric analysis of free hemoglobin (414 nm) as well as by quantification of the level of miR-451a relative to the level of miR-23a in our samples. Hence, the possibility of our findings being not a directional biological effect, but the product of noise variability is highly unlikely. Thus, given the hypothesis generating design, the miRNAs identified in our analysis might provide important hints towards adaptational processes despite their low dynamic.

Since different timepoints were defined for miRNA analysis, an influence by means of a circadian dynamic of miRNA expression must be considered. For two of the biomarkers mentioned above, a diurnal secretion pattern has been reported, miR-223-3p and miR-24-3p. For miR-223-3p peak-levels were observed at 11:38 a.m., for miR-24-3p peak levels were observed at 12:23 according to Heegaard et al.^25^. However, with regards to a diurnal secretion, it is important to note that the reported circadian changes in miRNA expression are low, and are by far surpassed by the changes observed following exposure to parabolic flight^25^. Thus, changes in miRNA expression during 24 h in normal conditions are unlikely as a relevant confounder^25^. Accordingly, the influence on the other two miRNAs miR-941 and miR-486-5p, which were not included in previous studies on diurnal expression changes is assumed to be negligible.

With regards to previous studies, miR-223-3p is known to be involved in myocardial injury and positively correlates with cardiac apoptosis and oxidative stress through interaction with krüppel-like factor 15 (KLF-15). Furthermore, an inverse correlation of miR-223-3p with an improved cell viability, prevention of cardiomyocyte apoptosis as well as with inhibition of reactive oxygen species and lipid peroxidation was shown in a former study^26^. Similar, elevated levels of miR-223-3p were reported under simulated microgravity in rats with an inhibitory effect on hepatocyte proliferation trough a cyclin dependent kinase 2/Cullin1 (CDK2/CUL1) signaling pathway^11^.

For miR-941, a significant upregulation in acute coronary syndrome was reported^27^. As miR-941 is associated with metabolic processes, inflammation and cell proliferation through its role in insulin-activated, mitogen-activated protein kinase (MAPK)—and T-cell receptor signaling, a connection with atherosclerosis was suggested as a potential explanation^27^. Additionally an involvement in proliferative processes and cell migration was reported in recent studies^28,29^. miR-941 expression levels have been shown to be elevated in cancer-derived cell lines and human embryonic stem cells and might also be involved in neuronal development^28,29^. A balancing effect of tumor suppressing P73 antisense RNA 1 T (TP73-AS1) by means of a miR-941 sponge was observed, emphasizing its proliferative aspect^28,29^.

miR-24-3p is known to be involved in cell proliferation and migration as well as inhibition of apoptosis in several malignancies such as lung cancer, hepatocellular carcinoma, and colon cancer^30–32^. Accordingly, miR-24-3p acts as a regulator of cell proliferation and apoptosis. Another study investigated the role of miR-24-3p in a murine cardiac ischemia-reperfusion model. A cardioprotective effect of miR-24-3p through an inhibition of receptor-interacting serine/threonine-protein kinase 1 (RIPK1) is suspected, correlating with a significant reduction in infarct size^33^. Furthermore, the same study showed an inverse correlation of miR-24-3p with the rate of apoptosis and the levels of Fas and tumor necrosis factor alpha (TNF-α)^33^.

miR-486-5p was investigated in various malignant diseases and mostly acts as a regulator of tumor-suppressor-genes. Negative regulations of NIMA-related kinase 2 (NEK2) in hepatocellular carcinoma and a repression of GRB2-associated-binding protein 2 (GAB2) in nonsmall cell lung cancer have been reported^34,35^. Besides its role in malignant processes, miR-486-5p was investigated in ischemic kidney injury and seems to attenuate ischemic effects in the kidney by targeting the phosphatase and tensin homolog (PTEN) and the Akt pathway^36^.

Regarding potential pathways involved in our findings discussed above, a target network analysis identified that genes from two KEGG pathways, the p53 signaling pathway and the cell cycle were highly enriched among the targets of the four microRNAs^37^. We observed that TP53 and E2F1 (miR-223-3p and miR-24-3p), and CDK4 (miR-24-3p and miR-486-5p) were targeted by two miRNAs. All other genes were targeted by only one miRNA. Accordingly, the interaction between the four miRNAs in the regulation of the p53 signaling pathway and the cell cycle are considered to be low. However, despite the high enrichment it must be pointed out that both pathways mentioned above are highly studied and annotated. Thus, they have a high likelihood of being linked with a selected group of miRNAs.

Interestingly, the findings of our study are in part consistent with former findings of a reduction in cell proliferation and a higher rate of apoptosis of in vitro and in vivo studies on changes in miRNA expression following exposure to microgravity. In vitro an altered expression pattern of miRNAs in human peripheral blood lymphocytes was shown to increase apoptosis and decrease cell proliferation following exposure to microgravity^38^. A recent study reported a decrease in TGF-Beta response and a change in miRNA signature as potential regulators of weightlessness induced changes in rodents. Above all, a reduction in cell proliferation and a higher rate of apoptosis was reported, matching prior in vitro studies^13^. Furthermore, an upregulation of miR-21 was reported to limit immune-response through a regulation of T-cell activation in microgravity^14^. A change in the expression pattern of immune-related miRNAs was also reported as a potential link to skeletal muscle atrophy in another investigation^39^. Of note, all studies mentioned above were conducted in cellular miRNAs.

Considering the novelty but also the hypothesis generating character of our study, we can only speculate about the potential adaptational processes following exposure to gravitational changes due to the hypothesis generating design of our study. Despite limited comparability with former studies, our results suggest an involvement in cardioprotective mechanisms as well as myocardial strain and the susceptibility for ischemic damage. Furthermore, cell-proliferation and apoptosis and possibly also the immune-system might be influenced by gravitational changes. As a rationale, a change in activation of DNA-repair mechanisms, a change in apoptotic processes and in the regulation of cell cycle progression and cell fate decision can be taken into consideration^40,41^. Potential implications of our findings are to be further validated and elucidated. However, analysis of the expression of circulating miRNAs might prove of further relevance for space medicine in the future, similar to the application of miRNA expression patterns in other extreme situations as for example in intensive care patients^42^.

With regards to the limitations of the present study, the hypothesis generating character must be emphasized. While parabolic flight was reported as a suitable space flight analog, the respective effects of microgravity and hypergravity cannot be distinguished properly in this study. Additionally, as we focused on circulating miRNAs, comparability with former studies conducted on cellular miRNAs is limited. Therefore, the results obtained must be interpreted with care. Additionally, a large part of research on miRNAs focuses on malignancies, thus complicating the interpretation of our results. On the other hand, the strength of the study is its novelty in a unique setting.

In conclusion, the present data indicate an alteration of expression patterns of circulating miRNAs following exposure to gravitational changes. We identified that genes from two KEGG pathways, the p53 signaling pathway and the cell cycle were highly enriched among the targets of the four microRNAs. This might suggest a change in cell cycle regulation as a potential explanation for weightlessness induced changes observed in former studies. Together, these findings point towards an adaption to gravitational changes already at the post-transcriptional level even after short term exposure, providing an important framework for future studies in this area.

## Methods

### Participants

Eight healthy participants were enrolled in this study. All voluntary participants were recruited and included at the University Clinic Düsseldorf after written informed consent. The study was conducted in accordance with the Declaration of Helsinki. The study protocol was approved by the German Ethics Committee (Medical Faculty of the University Hospital Duesseldorf, Germany) and by the French Ethics Committee (Medical Faculty of the University of Caen). The inclusion criteria were defined as: age >18 years; airworthiness; cardiorespiratory health; spontaneous circulation; signed informed consent. The exclusion criteria were defined as: history of primary cardiovascular and respiratory diseases or regular intake of medication except for oral contraceptives, missing or withdrawal of informed consent, insufficient requirements for airworthiness and pregnancy. Further details have been published in a study-outline paper^43^.

### Parabolic flight

The term “Parabolic flight” denominates a special aerial manoeuvre, in which the aircraft follows the trajectory of a parabola. By means of this manoeuvre, short phases of weightlessness and hypergravity can be induced. After starting at steady flight (1 g), an up to 47° ascent initiates a phase of hypergravity (1.8 g). After the climb, the plane is decelerated and directed into descent to follow the trajectory. In this state, a phase of weightlessness is achieved (0 g). With the plane tilting forward, an up to 45° descent is initiated, again inducing a phase of hypergravity (1.8 g), before re-entering the steady flight (1 g)^44^. Each of these phases averages 20–25 s, respectively.

### Set-up

The present study was performed in the course of the 31st Parabolic Flight Campaign (PFC) of the German space agency (Deutsches Zentrum für Luft- und Raumfahrt - DLR) conducted by the company NoveSpace, which took place from February 26th to March 11th 2018, in Bordeaux, France. The aircraft used in this campaign was a modified Airbus A-310. The PFC consisted of four flight days, with 31 parabolic flight manoeuvres per flight day. Each study subject participated in one complete flight day. In all participants serum samples were withdrawn before entering the plane 1 h prior parabolic flight (baseline 08:00 ± 0.75 h), 1 h after parabolic flight (13:00 ± 1.0 h) and 24 h after parabolic flight 08:00 ± 0.75 h). At each of the time points, serum samples were collected with individual punctures. For each blood withdraw, the following materials were used: BD vacutainer tubes (Becton Dickinson, Mountain View, CA), 3 SST tubes (Reference # 367957, Tube size: 75 × 13 mm, draw volume: 3.5 ml), 1 SST tube (Reference # 367955, Tube size: 100 × 13 mm, Draw volume: 5 ml. After completion of each blood-draw, blood was stored at room-temperature for 3 min. Afterwards, centrifugation at 2000×*g* for 7 min was conducted. Following centrifugation, serum samples aliquots were frozen and stored at −80 °C until further analysis.

### Total RNA extraction

Serum samples were thawed at room temperature followed by centrifugation at 12,000×*g* for 5 min at 4 °C to remove any cellular debris. Extraction was conducted by use of the miRNA easy QIAgen kit. For homogenization, 200 µl of serum were mixed with 1000 µl Qiazol and 1 µl of a mix of three synthetic spike-in controls (Qiagen, Germany). After a 10-min incubation at room temperature, 200 µl chloroform were added to the lysates followed by cooled centrifugation at 12,000×*g* for 15 min at 4 °C. Precisely 650 µl of the upper aqueous phase were mixed with 7 µl glycogen (50 mg/ml) to enhance precipitation. Samples were transferred to a miRNeasy mini column, and RNA was precipitated with 750 µl ethanol followed by automated washing with RPE and RWT buffer in a QiaCube liquid handling robot. Finally, total RNA was eluted in 30 µl nuclease free water and stored at −80 °C until further analysis.

### Small RNA sequencing

Equal volumes of total RNA (2 µl) were used for small RNA library preparation using the CleanTag smallRNA library preparation kit (TriLink Biotechnologies, US). Homogeneity of RNA extraction efficiencies across all samples was confirmed by RT-qPCR (Supplement Fig. 2B). Adapter-ligated libraries were amplified using barcoded Illumina reverse primers in combination with the Illumina forward primer. Twenty-four PCR cycles were used for library preparation according to the manufacturers recommendation for low RNA input samples. A pool consisting of all 24 sequencing libraries was prepared by mixing samples at equimolar rates on the basis of a DNA-1000 high-sensitivity bioanalyzer results (Agilent, CA). The DNA library pool was gel-purified to enrich for microRNAs with an insert size of 18–24 nt, corresponding to a library size of approximately 145 bp (Supplement Fig. 2B).

Sequencing was performed on an Illumina HiSeq 2500 with 50 bp single-end runs. Overall quality of the next-generation sequencing data was evaluated automatically and manually with fastQC v0.11.8^45^ and multiQC v1.7^46^. Reads from all passing samples were adapter trimmed and quality filtered using cutadapt v2.3^47^ and filtered for a minimum length of 17 nt. Mapping steps were performed with bowtie v1.2.2^48^ and miRDeep2 v2.0.1.2^49^, whereas reads were mapped first against the genomic reference GRCh38.p12 provided by Ensembl^50^ allowing for two mismatches. Those reads showing a genome alignment were subsequently mapped against miRbase v22.1^51^, filtered for miRNAs of hsa only, allowing for one mismatch. For a general RNA composition overview, nonmiRNA mapped reads were mapped against RNAcentral^52^ and then assigned to various RNA species of interest.

Raw and normalized data were uploaded to NCBI Gene Expression Omnibus^53^ and are accessible through GEO Series accession number GSE147380.

### Assessment of hemolysis

As lysis of red blood cells (hemolysis) could confound the analysis of circulating microRNAs due to the release of miRNAs from red blood cells, an assessment of hemolysis was conducted^54^. In order to determine the degree of hemolysis two methods were applied: First, 2 µl of serum were loaded on a NanoDrop spectrophotometer to determine absorbance at 414 nm (free hemoglobin). Secondly, miR-23a-3p and miR-451a were analyzed by RT-qPCR and the hemolysis miRNA ratio according to Blondal et al.^23^ was calculated: ΔCq = CqmiR-23a-3p – CqmiR-451a.

### Reverse transcription and qPCR (RT-qPCR)

The identical RNA samples that were used for NGS analysis, were used for results verification by RT-qPCR. Starting from total RNA samples, cDNA was synthesized using the miRCURY RT Kit (Qiagen, Germany). Reaction conditions were set according to recommendations by the manufacturer. In total, 2 µl of total RNA were used per 10 µl reverse transcription (RT) reaction. To monitor RT efficiency and presence of impurities with inhibitory activity, a synthetic RNA spike-in (cel-miR-39-3p) was added to the RT reaction. Validated LNA-enhanced forward and reverse miRCURY primer assays for all targets, including spike-in controls, were obtained from Qiagen. PCR amplification was performed in a 96-well plate format in a Roche LC480 II instrument (Roche, Germany) using miRCURY SYBR® Green mix (Qiagen, Germany) with the following settings: 95 °C for 10 min, 45 cycles of 95 °C for 10 s, and 60 °C for 60 s, followed by melting curve analysis. To calculate the cycle of quantification values (*Cq*-values), the second derivative method was used. *Cq*-values were normalized to the RNA spike-in control level, by subtracting the individual miRNA *Cq*-value from the RNA Spike-In Cq, thus obtaining delta-Cq (Δ*Cq*) values that were used for the analysis.

### Target network analysis

Target network analysis was conducted using miRNet (www.mirnet.ca), a public and web-based tool that allows to visualize the intersections between individual miRNA regulatory functions based on miRNA-target interaction data from 11 databases^55^. miRbase miRNA IDs for the differentially regulated miRNAs were uploaded. Since cell-free circulating miRNAs were analyzed no tissue background was selected (“exosomes” were not selected since most miRNAs in serum are in protein complexes). A “hypergeometric test” and “empirical sampling” were performed to identify gene pathways enriched among the identified targets.

### Statistics

Statistical analysis of preprocessed NGS data was done with R v3.6 and the packages pheatmap v1.0.12, pcaMethods v1.78 and genefilter v1.68. Differential expression analysis with edgeR v3.28^56^ used the quasi-likelihood negative binomial generalized log-linear model (GLM) functions provided by the package. FDR correction was performed to adjust for multiple testing, and a cut-off of FDR < 5% was applied^57^. No paired testing model in EdgeR was applied. Significance of differentially regulated miRNAs (according to EdgeR) was determined by using EdgeR GLM method together with Benjamini–Hochberg adjustment for multiple testing. An FDR of <5% was considered statistically significant. Repeated measures ANOVA analysis combined with Tukey’s multiple comparison test was used to assess RT-qPCR data. Regarding baseline characteristics and laboratory parameters, analysis was conducted using SPSS 25 (IBM SPSS Statistics, USA). Non-normally distributed data are given as median + interquartile range (IQR). Categorical data are given in number (%). Correlation analysis were performed using Spearman’s rank correlation coefficient. A *p*-value < 0.05 was considered statistically significant for comparison of baseline characteristics and correlation analysis. All tests were two-sided.

### Reporting summary

Further information on research design is available in the Nature Research Reporting Summary linked to this article.

## Supplementary information

Supplementary Information

Reporting Summary

## Data Availability

The datasets of raw and normalized generated and analysed during the current study are were uploaded to NCBI Gene Expression Omnibus^53^ and are accessible through GEO Series accession number GSE147380.
