# Supplementary material for: Next-generation sequencing analysis of circulating micro-RNA expression in response to parabolic flight as a spaceflight analogue
Source: NPJ Microgravity. 2020 Nov 2;6:31. doi: 10.1038/s41526-020-00121-9 (PMC7606465; doi:10.1038/s41526-020-00121-9)
Supplement: Supplementary file 1 — Supplementary Information [file 41526_2020_121_MOESM1_ESM.pdf]

Supplement Figure/Table Legends:

Supplement Figure 1: Hemolysis quality control. a) RNA and cDNA spike-in controls were used to determine the robustness (RNA extraction efficiency and enzyme inhibition) of RT-qPCR analysis. b) Hemolysis miRNA ratio calculated as CqmiR-23a-3p – CqmiR-451a. c) Absorbance at 414nm (OD414). d) Distribution of hemolysis miRNA ratio, hemolysis OD414, miR-23a-3p and miR-451a across the three experimental groups.

Supplement Figure 2: RNA recovery and quality control of NGS libraries. a) Confirmation of RNA recovery from serum samples based on RNA spike-in levels. b) Quality control of NGS library pool before (left) and after size selection of microRNA inserts (right)

Supplement Figure 3: Quality control of NGS data. a/b) Mean and per sequence quality (phred) scores after adapter trimming. c) Read size distribution. d/e) Absolute and relative read classification. f) Numerical value for read classification. g) Number of distinct microRNAs (microRNA diversity) per sample showing an abundance of TPM>1, TPM>5 and TPM>10.

Supplement Table 1: List of all miRNAs with TPM >5 that were identified at each time point for each sample

Supplement Table 2-5: Correlation analysis of miRNA-expression and baseline characteristics/laboratory parameters

Supplement Table 6: KEGG enrichment details

Supplement Table 7a/7b: Genes regulated by at least one of the four differentially regulated miRNAs

Supplement Figure 1

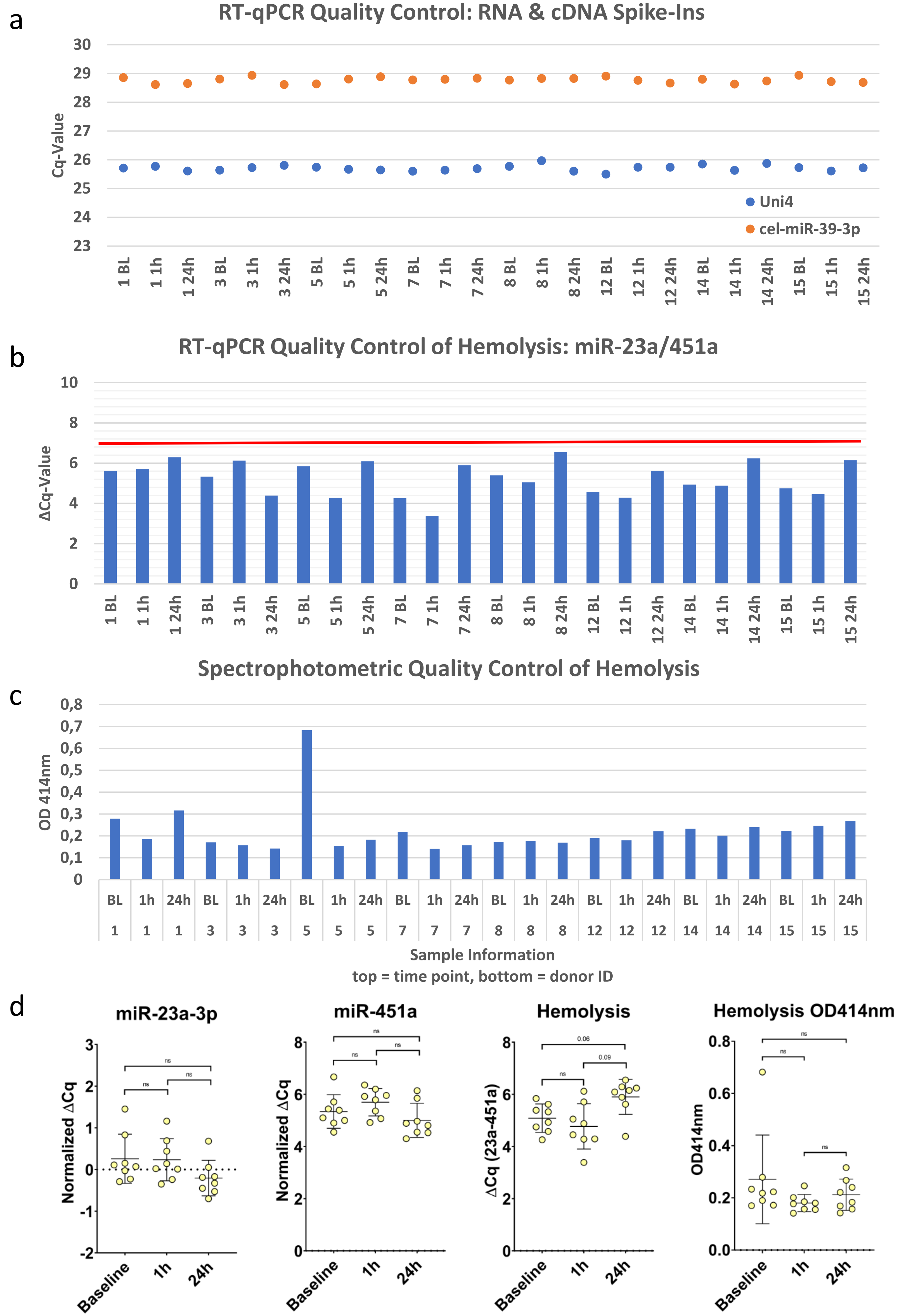

Supplement Figure 2

a

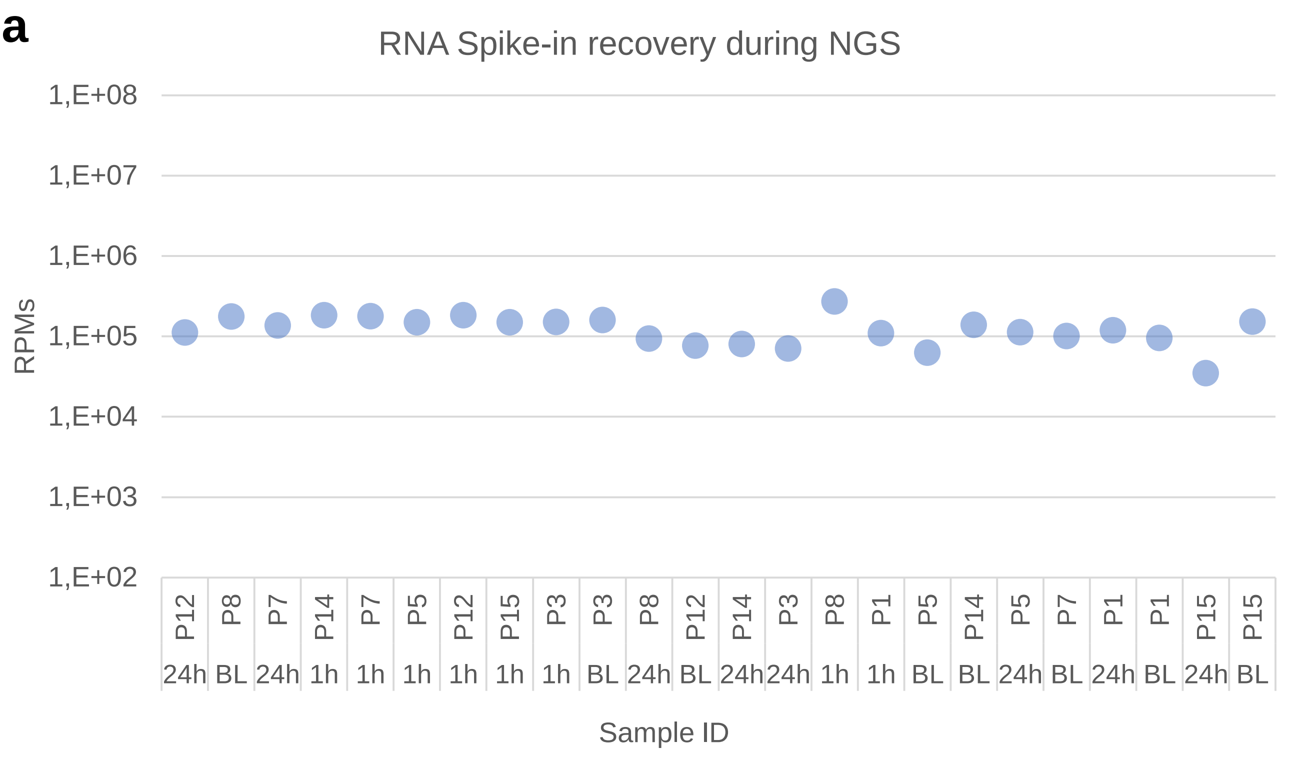

b

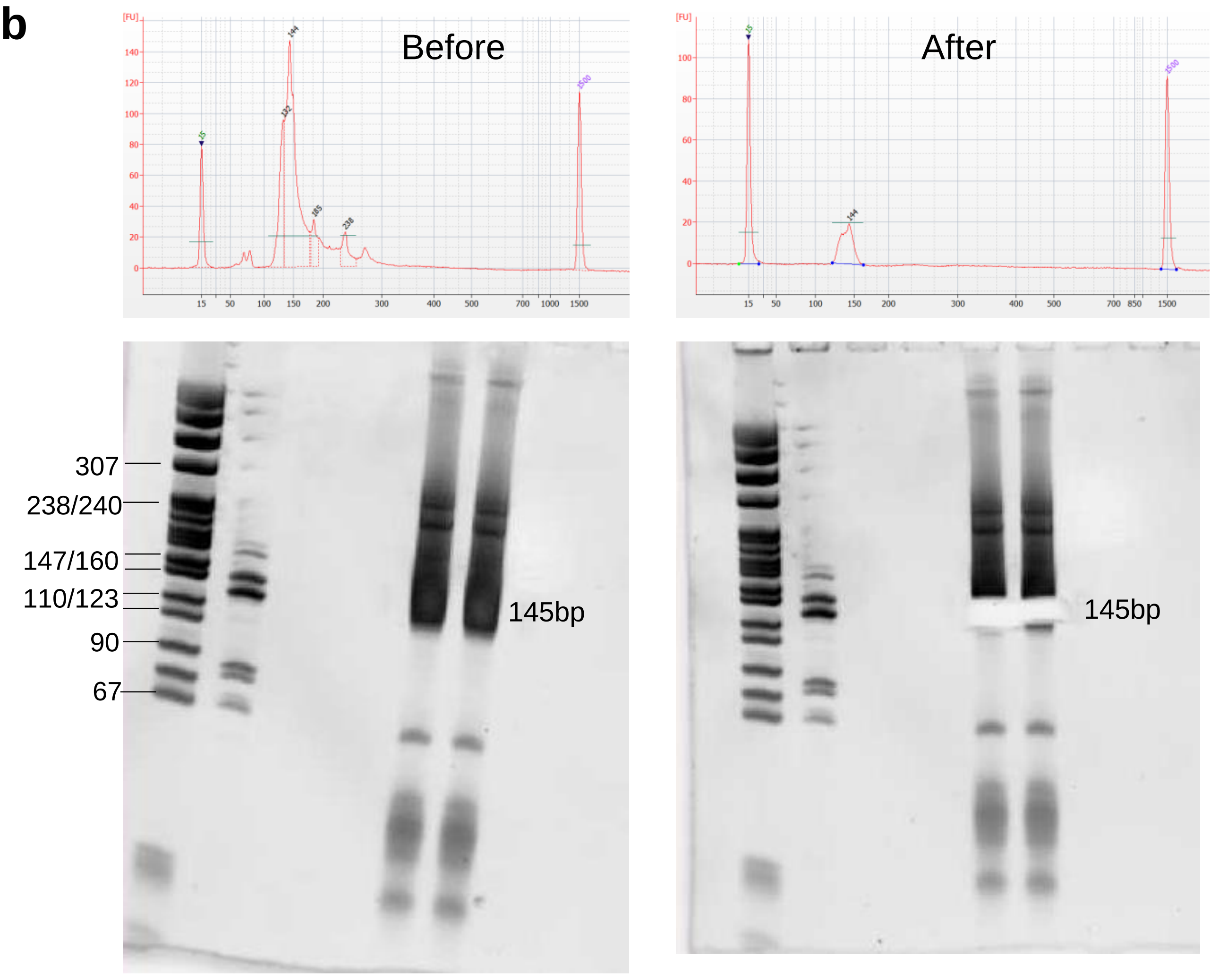

Supplement Figure 3

a

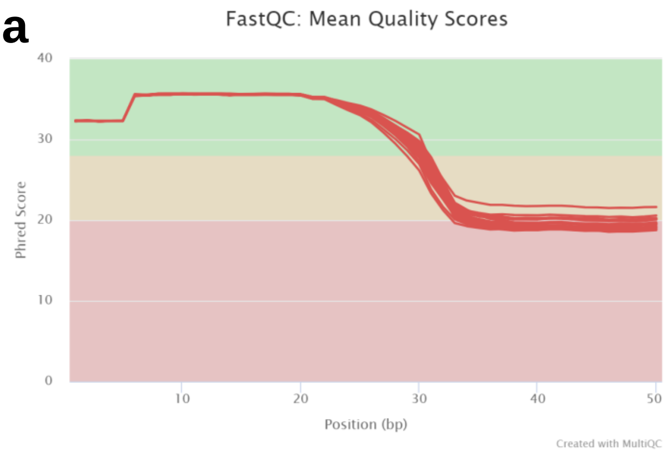

b

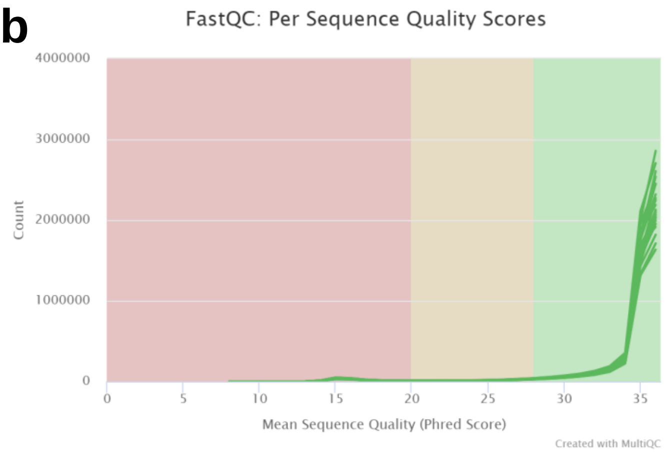

c

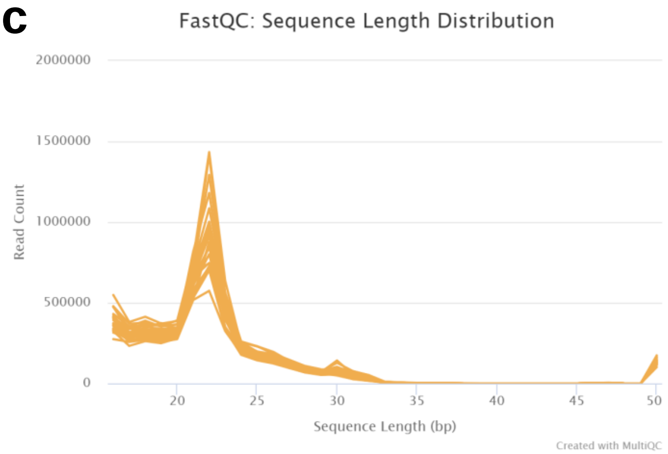

d

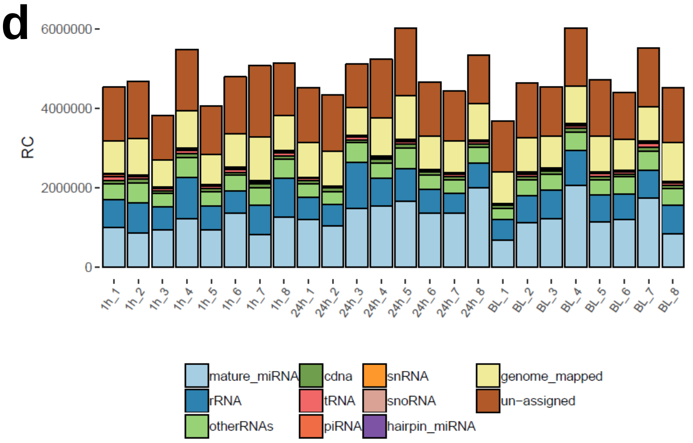

e

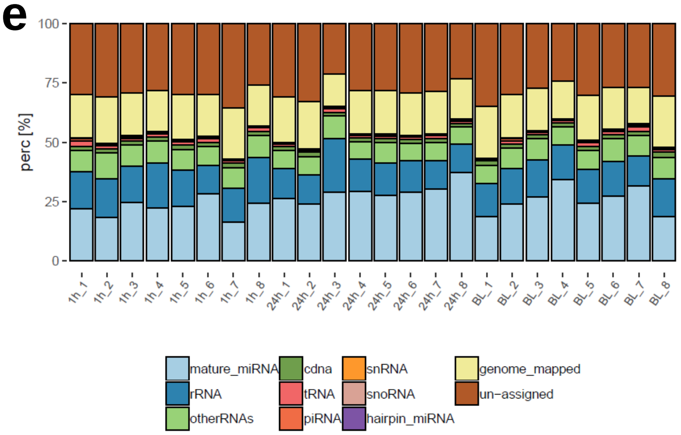

f

sample	mature_miRNA [#]	mature_miRNA [%]	rRNA [#]	rRNA [%]	otherRNAs [#]	otherRNAs [%]	cdna [#]	cdna [%]	tRNA [#]	tRNA [%]
**1h**										
1h_1	994990	21.93	710652	15.67	400661	8.83	80963	1.78	98928	2.18
1h_2	865665	18.47	756979	16.15	508387	10.84	88623	1.89	56394	1.20
1h_3	945871	24.78	575666	15.08	342428	8.97	64470	1.69	44196	1.16
1h_4	1229994	22.42	1030779	18.79	506795	9.24	95489	1.74	79561	1.45
1h_5	938311	23.12	608297	14.99	362195	8.93	72193	1.78	50946	1.26
1h_6	1360112	28.38	561464	11.71	392320	8.19	77044	1.61	73340	1.53
1h_7	822070	16.19	736655	14.51	434533	8.56	101825	2.01	50696	1.00
1h_8	1254062	24.36	987079	19.17	481780	9.36	82449	1.60	80497	1.56
**24h**										
24h_1	1195726	26.41	569878	12.59	343267	7.58	78641	1.74	45288	1.00
24h_2	1041108	23.96	539986	12.42	330095	7.60	81950	1.89	30569	0.70
24h_3	1480674	28.96	1156794	22.63	494701	9.68	66465	1.30	78436	1.53
24h_4	1536782	29.35	714388	13.64	379793	7.25	86723	1.66	50487	0.96
24h_5	1663103	27.64	816143	13.56	521733	8.67	106525	1.77	62666	1.04
24h_6	1355014	29.07	611293	13.12	346300	7.43	78877	1.69	43454	0.93
24h_7	1351489	30.43	517847	11.66	342143	7.70	74657	1.68	58113	1.31
24h_8	2001861	37.41	624907	11.68	391774	7.32	83496	1.56	51758	0.97
**BL**										
BL_1	688355	18.68	515637	14.00	282495	7.67	69534	1.89	22132	0.60
BL_2	1113191	24.03	686802	14.83	398373	8.60	79717	1.72	68946	1.49
BL_3	1227752	26.99	711545	15.64	402089	8.84	75098	1.65	44964	0.99
BL_4	2063281	34.24	879527	14.60	459840	7.63	95059	1.58	65936	1.09
BL_5	1142683	24.16	678361	14.35	379835	8.03	82718	1.75	68822	1.46
BL_6	1208484	27.44	635599	14.43	429365	9.75	73712	1.67	53919	1.22
BL_7	1748827	31.63	701461	12.69	473538	8.56	88358	1.60	106971	1.93
BL_8	839508	18.57	721117	15.95	415335	9.19	94710	2.10	52394	1.16

g

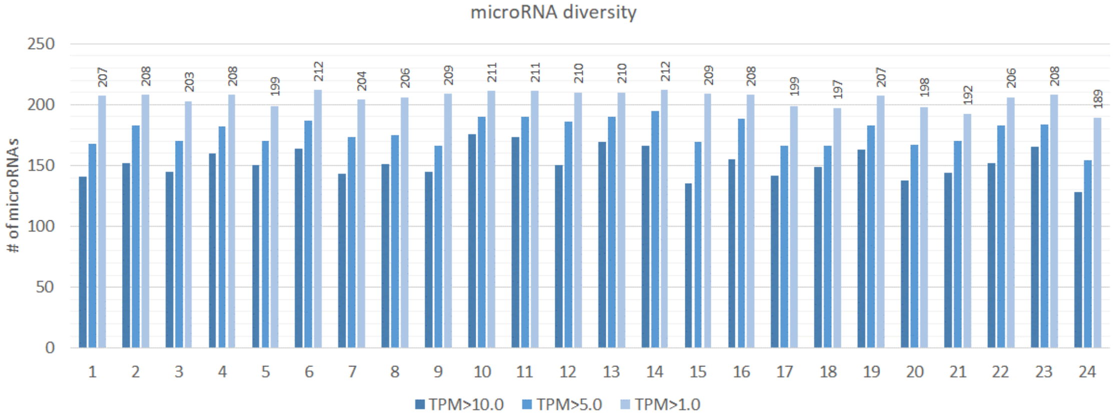

Supplement Table 1

microRNA ID	p77005	p77013	p77015	p77020	p77021	p77023	p77025	p77027	p77007	p77008	p77009	p77010	p77011	p77012	p77018	p77019	p77004	p77006	p77014	p77016	p77017	p77022	p77024	p77026
Donor ID	P8	P3	P12	P5	P14	P7	P1	P15	P14	P7	P5	P12	P15	P3	P8	P1	P12	P7	P8	P14	P3	P5	P1	P15
Timepoint	0h	0h	0h	0h	0h	0h	0h	0h	1h	1h	1h	1h	1h	1h	1h	1h	24h	24h	24h	24h	24h	24h	24h	24h
Sex	female	male	female	male	female	female	male	male	female	female	male	female	male	male	female	male	female	female	female	female	male	male	male	male
hsa-miR-98-5p	73,28	120,45	100,48	293,07	113,98	439,85	55,16	56,41	80,46	372,22	318,53	120,84	167,34	145,63	75,04	398,92	75,97	111,37	751,52	72	66,81	140,96	91,87	40,17
hsa-miR-6131	10,04	10,79	16,05	12,94	12,05	22,48	14,29	11,95	17,64	31,36	15,46	18,04	16,27	13,35	8,67	14,18	11,26	5,98	23,86	10,89	11,63	6,65	8,33	7,66
hsa-miR-148a-5p	1,63	13,38	23,09	14,11	19,24	21,57	35,45	15,93	22,04	30,08	26,2	23,51	14,54	16,69	5,32	12,24	4,64	11,5	8,02	12,41	53,68	9,23	18,46	16,82
hsa-miR-185-5p	48,04	45,55	35,84	94,59	59,85	47,23	15,19	29,42	29,98	35,62	71,77	48,66	35,73	79,07	34,27	41,95	28,05	29,91	103,67	59,78	31,74	66,72	56,52	18,31
hsa-miR-150-3p	8,96	25,04	21,77	21,08	7,82	16,8	22,06	43,58	6,61	6,83	41,65	8,57	37,21	13,56	15,17	102,93	11,04	19,33	64,16	14,13	24,26	17,38	17,11	35,13
hsa-miR-342-3p	13,3	21,37	19,13	19,75	14,59	34,74	30,2	23,45	10,58	27,94	58,94	12,58	29,33	10,64	17,14	108,95	23,19	16,11	59,07	8,59	19,78	22,31	35,58	16,07
hsa-miR-7-5p	15,47	26,55	8,57	27,38	23,26	25,43	12,66	15,49	11,02	25,17	38,77	17,68	17	42,56	21,86	8,55	17,45	25,77	41,66	39,15	19,94	22,96	13,51	19,43
hsa-miR-144-3p	152	215,65	132,8	369,74	121,17	208,91	178,69	129,86	127,64	123,08	198,04	145,08	174,48	525,34	198,35	260,64	145,09	207,77	393,17	148,01	105,36	420,72	387,28	179,76
hsa-miR-26b-5p	423,15	1085,81	559,98	955,71	748,39	1509,63	291,36	458,39	525,54	1490,16	1421,87	774,62	749,69	1422,05	529,45	1300,47	442,35	669,34	1587,15	540,46	350,16	936,92	827,7	255,06
hsa-miR-181c-5p	51,57	71,24	58,48	65,22	51,39	149,65	79,4	23,01	42,77	138,86	83,3	68,35	87,98	47,36	32,3	56,52	9,28	21,17	35,4	38,19	75,28	29,61	47,06	29,71
hsa-miR-1224-5p	2,17	6,48	3,08	6,97	2,11	1,36	7,05	0,22	3,53	1,28	14,41	6,2	1,48	5,42	3,74	0,78	3,09	10,12	3,91	5,73	3,66	17,59	2,03	3,36
hsa-miR-93-5p	310,51	621,91	213,92	801,54	582,39	710,76	372,75	263,71	358,44	593,63	783,76	548,06	381,99	870,63	358,87	662,66	305,2	394,61	1374,53	412,7	219,2	737,82	631,59	181,63
hsa-miR-148a-3p	343,62	1167,41	506,78	1039,52	492,94	1153,34	1254,24	756,16	488,95	1207,53	1414,8	982,03	1016,59	738,36	394,72	686,16	372,78	269,9	312,97	380,99	878,13	526,28	939,83	548,24
hsa-miR-185-3p	2,99	7,99	2,86	14,44	0	11,13	5,43	6,42	4,41	21,12	12,31	7,11	10,1	5,22	8,86	9,52	5,52	2,53	11,74	3,82	0	9,65	5,18	11,21
hsa-miR-19b-3p	12,76	18,56	11,65	31,03	17,76	37,24	10,13	15,93	6,61	32,85	22,27	14,58	16,51	27,33	7,68	22,72	8,39	13,58	28,95	14,51	17,78	23,6	13,28	6,54
hsa-miR-1260b	6,79	11,23	4,18	3,32	2,75	11,35	3,44	2,88	1,32	12,16	7,07	14,95	6,41	8,55	5,52	9,13	8,61	1,38	12,32	0	4,99	6,44	0,9	2,62
hsa-miR-1260a	2,99	2,16	0	0	2,75	6,81	1,81	11,5	1,32	12,37	9,69	5,29	4,68	8,14	8,47	10,29	1,99	2,76	3,52	2,67	10,97	1,5	7,43	0
hsa-miR-122-5p	595,78	4092,41	729,93	3033,42	354,64	644,68	5147,19	2938,81	779,93	590,86	3077,66	1017,21	3697,68	3413,26	604,49	3392,16	1508,57	866,53	355,81	1077,67	1238,93	1706,28	4893,49	1042,11
hsa-let-7c-5p	127,03	270,48	139,83	218,23	136,19	168,95	414,89	222,34	188,92	167,87	176,56	161,48	325,06	263,71	146,94	287,83	140,01	177,86	187,98	146,29	158,54	181,29	290,91	117,16
hsa-miR-483-5p	18,46	64,54	15,17	59,08	9,73	35,2	117,38	91,37	16,31	26,24	74,92	28,8	92,91	50,7	15,95	62,34	26,28	25,31	10,17	15,09	32,24	36,69	81,96	63,53
hsa-miR-126-3p	11,67	16,84	16,71	30,37	19,46	35,42	18,81	42,48	23,15	33,28	30,39	15,49	34,26	24,2	7,88	17,09	10,6	17,95	22,89	1,72	16,45	21,45	15,99	8,97
hsa-miR-127-3p	60,53	18,13	52,99	18,42	6,77	146,24	44,31	14,16	20,28	87,24	26,98	11,48	16,76	55,91	32,3	39,23	50,79	34,51	73,16	17,57	114,17	5,15	70,25	16,07
hsa-miR-4286	1,36	7,34	3,3	5,64	9,09	19,53	7,23	4,2	2,87	24,1	13,36	13,12	7,89	7,3	4,92	8,93	0,66	4,14	4,3	3,82	5,32	2,79	6,3	0
hsa-miR-130a-3p	26,06	35,83	11,65	23,07	16,92	66,31	20,26	27,87	18,08	20,9	40,6	10,21	31,55	38,18	26,2	21,95	39,97	23,93	34,82	20,63	20,61	33,04	55,62	29,52
hsa-miR-361-5p	17,64	41,66	12,97	40,66	25,8	25,43	17,18	21,46	18,3	71,46	63,92	34,63	36,97	35,89	26,98	23,31	13,69	16,34	33,25	20,05	14,29	28,75	25,89	5,42
hsa-miR-4710	6,79	3,67	0	3,65	0	4,77	15,01	6,19	3,97	4,91	3,14	4,92	1,97	8,14	7,09	1,75	14,35	11,73	0,98	8,98	0	7,51	1,13	5,23
hsa-miR-5010-5p	8,69	4,96	17,37	5,64	0	1,36	4,16	2,65	2,2	3,2	6,02	6,93	12,08	6,05	4,33	1,36	5,3	3,68	2,93	6,49	0	4,29	0,23	2,43
hsa-miR-873-3p	3,26	3,67	1,1	2,32	1,9	4,09	12,12	7,08	1,98	5,55	3,67	3,1	2,71	7,93	5,32	1,17	9,94	0	3,33	4,97	23,27	2,79	1,35	10,09
hsa-miR-484	41,8	33,89	28,14	54,76	48,64	79,71	59,32	40,04	47,4	61,43	73,35	55,41	35,73	86,17	31,91	55,93	47,04	37,05	64,35	46,6	27,25	64,36	94,34	22,24
hsa-miR-99a-5p	16,01	29,36	23,31	19,58	20,51	28,16	79,4	28,76	13,23	38,4	42,7	20,05	42,14	24,41	16,94	25,44	20,1	15,19	6,26	6,88	38,56	30,25	37,6	22,8
hsa-miR-425-5p	162,86	337,18	89,92	465,33	241,92	391,03	216,3	215,92	188,48	482,29	469,68	303,65	345,52	461,29	186,92	281,03	176,67	167,28	339,96	234,52	176,32	366,66	507,52	168,36
hsa-miR-3615	39,9	128,87	59,36	107,37	137,03	163,72	27,49	58,4	70,76	224,61	186,51	133,05	131,6	97,02	38,01	69,72	56,98	32,44	89,39	61,11	24,6	59,43	66,87	36,06
hsa-miR-361-3p	2,99	16,41	3,52	13,61	17,13	12,26	6,87	15,04	5,07	34,34	33,79	12,39	5,42	12,73	4,33	27,19	7,51	2,3	38,73	6,68	5,15	9,65	20,26	7,1
hsa-miR-30d-5p	809,93	1367,52	582,85	1434,81	891,77	1803,7	1092,19	1016,99	780,59	1466,48	1869,55	939,38	1451,82	1615,87	720,7	1314,64	669,37	900,82	1178,53	723,03	747,18	1385,32	1429,79	704,83
hsa-miR-107	411,21	793,74	359,25	922,86	365,42	555,66	878,96	605,28	453,45	359,21	687,88	340,28	832	1095,75	361,04	759,58	353,57	495,16	636,11	428,36	713,77	953,44	922,95	1004,36
hsa-miR-3960	11,67	28,28	27,92	7,97	21,57	14,31	37,26	5,75	23,15	9,17	12,57	25,52	11,83	20,45	10,24	13,98	16,12	19,1	15,26	29,22	3,99	24,24	17,79	6,91
hsa-miR-3168	18,19	21,15	28,58	15,1	61,54	24,07	204,01	11,5	27,34	15,36	25,41	78,92	24,89	39,85	21,67	53,8	64,93	56,14	36,38	22,54	13,13	32,18	22,74	85,58
hsa-miR-425-3p	15,74	38,64	14,73	33,19	18,82	39,97	14,47	15,49	16,97	46,71	41,65	26,61	38,69	35,26	7,68	19,62	22,97	3,45	23,28	17,76	24,76	24,46	20,26	16,07
hsa-miR-130b-3p	36,37	26,12	20,67	77,83	28,34	26,11	24,96	28,54	22,26	27,3	38,24	19,68	38,94	28,17	20,68	29,33	30,92	20,02	33,64	27,5	21,27	44,84	24,99	11,96
hsa-miR-1307-5p	4,34	9,93	0	8,3	1,27	6,13	8,86	8,85	2,65	6,83	17,29	8,57	10,6	9,6	2,17	3,69	0,88	0	2,74	3,63	13,63	18,88	2,03	3,18
hsa-miR-769-5p	11,4	31,52	16,27	32,03	20,94	32,47	7,78	14,38	17,64	48,85	66,54	24,42	27,36	12,1	6,89	20,78	10,82	14,73	12,13	4,01	30,58	10,08	14,86	18,5
hsa-miR-100-5p	31,49	36,91	33,42	46,13	35,74	32,25	81,57	53,32	21,16	32,42	56,58	46,29	65,55	64,89	45,7	40,4	28,49	31,98	16,63	8,59	67,31	51,71	43,68	40,74
hsa-miR-152-3p	4,34	9,71	5,5	12,94	5,92	28,84	13,2	7,74	3,31	13,22	11,79	5,29	12,08	6,88	2,36	7,77	4,42	14,96	17,41	9,36	15,12	14,59	14,41	10,84
hsa-miR-10a-5p	521,14	477,93	538,44	401,77	327,57	589,27	601,53	760,36	648,77	658,48	680,29	561,37	828,31	427,49	596,22	417,37	498,22	705,01	288,32	351,78	551,91	556,1	497,61	529,93
hsa-miR-92b-5p	8,41	3,67	4,4	1,66	11,21	0,45	5,97	0,22	6,17	5,12	1,57	5,47	7,39	5,22	3,35	4,27	0	0	11,35	4,97	5,98	3,65	6,75	3,55
hsa-miR-222-3p	63,51	90,23	34,74	152,01	42,08	269,09	52,63	68,8	68,56	196,88	225,8	56,5	130,86	106,82	69,92	115,95	58,52	59,13	107,19	41,44	62,49	92,9	118,44	16,07
hsa-miR-1307-3p	39,9	86,13	48,37	95,59	64,5	128,75	63,3	50	71,2	101,53	141,72	33,9	97,59	69,27	22,45	87,59	68,02	42,34	48,31	40,87	55,01	67,58	50,21	41,67
hsa-miR-182-5p	123,23	545,71	516,01	305,85	749,24	450,53	333,68	151,1	422,81	310,15	298,62	541,32	295,98	791,77	227,69	560,89	348,05	413,25	797,1	586,1	270,39	416,86	463,84	324,76
hsa-miR-421	21,99	37,99	29,46	52,27	61,96	64,26	9,04	31,41	47,18	48,42	75,18	49,58	28,09	30,88	31,32	26,41	34,23	19,79	41,27	22,73	29,58	55,14	28,6	23,92
hsa-miR-193b-5p	9,23	13,38	4,4	2,82	0	8,4	38,34	11,06	8,6	2,13	5,5	6,2	21,44	5,42	9,06	10,1	12,37	2,53	0,78	3,44	1,5	9,01	23,87	4,11
hsa-miR-374a-5p	1,9	2,37	4,18	6,47	1,9	9,76	9,22	1,55	0	3,63	9,69	5,65	4,68	6,05	3,94	10,1	0	0	6,26	2,48	7,81	11,37	4,73	1,87
hsa-miR-141-3p	42,07	46,63	69,48	198,48	61,54	76,3	79,76	65,26	73,85	101,53	240,21	41,74	83,3	13,98	27,97	75,74	126,54	101,47	39,32	51,18	23,76	219,27	52,01	67,83
hsa-miR-1-3p	11,94	4,96	3,96	7,63	0,42	3,63	0	13,49	3,53	7,68	12,84	11,12	24,15	7,93	12,8	4,86	0,88	2,53	12,71	0,57	2,83	5,15	0,68	3,92
hsa-let-7e-5p	54,29	103,83	54,53	78,16	63,44	246,84	56,97	95,79	76,49	154,01	136,74	54,86	135,79	78,24	66,18	63,51	49,47	69,03	74,92	48,51	31,24	42,91	63,27	47,09
hsa-miR-760	4,89	5,83	2,64	6,14	3,59	12,94	4,16	11,28	0	3,41	4,98	8,38	0,99	4,59	3,35	6,99	1,77	4,14	3,91	4,77	1,83	3,22	7,66	0
hsa-miR-939-5p	0,54	4,1	6,6	4,48	0	2,27	0	0	3,53	1,49	2,62	1,64	2,71	5,01	3,55	2,91	3,31	2,76	1,76	0,19	3,16	0,86	1,58	0
hsa-miR-143-3p	339,55	1237,57	1028,72	1508	1148,71	1798,93	1022,93	639,79	1098,47	2520,86	2799,73	1570,2	1373,94	807,42	620,64	713,94	413,86	443,62	203,82	605,39	1415,25	482,51	683,37	501,34
hsa-miR-20a-5p	12,76	19,21	2,42	8,3	11,84	14,99	0	11,28	13,67	13,65	19,12	9,48	18,24	20,45	7,88	10,29	4,86	17,72	14,47	7,07	5,15	12,87	23,19	2,43
hsa-miR-1290	66,23	523,48	550,75	340,2	311,07	1015,27	187,55	193,58	180,1	1034,32	757,56	498,85	436,46	217,19	150,09	177,51	130,3	92,73	139,47	163,67	405,66	88,61	158,29	156,96
hsa-miR-1908-5p	1,9	17,92	7,04	5,81	7,61	34,97	6,87	11,5	5,29	34,34	9,17	12,03	11,09	16,27	2,56	9,32	13,69	13,58	12,71	4,58	5,65	6,22	18,69	5,23
hsa-miR-1255b-5p	1,9	3,89	2,42	4,31	7,61	2,27	2,35	1,55	1,98	2,56	2,88	0	1,73	1,25	0,59	0,58	2,65	0	5,09	4,58	8,14	6,44	0	0
hsa-miR-363-3p	84,14	164,49	40,23	263,03	113,35	117,17	139,8	90,04	55,55	64,21	190,96	53,59	124,21	317,12	99,47	161	61,62	104,23	199,71	91,09	116,66	337,7	232,82	141,08
hsa-miR-451a	1157,09	1980,8	525,24	2412,77	1234,78	1639,29	1183,53	1166,98	755,24	859,84	1407,73	904,02	1320,71	3730,8	1049,83	1760,95	931,07	1563,49	2638,34	1368,14	579,16	2159,62	3182,69	899,16
hsa-miR-625-3p	8,14	2,81	17,59	24,06	15,44	7,95	4,52	4,42	7,27	18,56	21,22	11,85	10,84	11,06	5,12	6,6	16,34	6,9	18,78	7,26	10,3	9,65	6,75	0
hsa-miR-146a-5p	361,54	341,5	286,7	457,86	299,87	735,51	295,52	723,64	359,1	566,54	625,28	275,58	925,41	436,46	270,43	798,42	340,76	381,04	706,53	206,63	326,39	590,86	444,7	315,79
hsa-miR-4516	40,71	63,68	51,89	102,72	74,86	80,39	108,69	25,66	106,7	165,31	113,95	48,3	62,35	120,17	50,03	90,89	22,75	53,84	54,97	58,06	53,51	86,68	37,6	40,36
hsa-miR-1273h-3p	4,89	6,91	0	3,48	0,85	10,9	2,53	10,18	1,98	17,06	9,95	13,67	14,79	10,22	4,73	8,74	2,87	3,22	6,26	1,72	12,63	1,29	4,73	0
hsa-miR-4508	76,27	46,63	43,09	89,61	127,52	168,04	100,01	46,02	147,92	55,03	64,96	129,04	59,64	145	129,01	85,84	76,63	173,26	164,31	166,53	13,79	78,95	70,93	66,9
hsa-miR-146b-5p	534,71	409,5	494,46	446,08	641,18	919,9	627,03	274,55	611,29	678,31	512,11	265,01	504,97	573,12	436,28	2192,88	505,51	462,49	2468,36	441,72	977,85	615,75	395,39	362,88
hsa-miR-106b-5p	7,87	22,23	13,85	9,63	12,27	6,81	7,6	4,42	4,41	10,24	15,46	7,11	20,21	23,99	8,86	15,34	23,19	3,68	25,04	11,46	0	21,88	23,87	3,55
hsa-miR-21-3p	15,74	8,2	12,97	13,77	12,69	25,89	9,22	10,4	29,1	12,16	24,62	8,75	30,81	9,39	4,73	14,57	0	16,34	12,52	11,46	10,3	40,55	12,61	16,26

hsa-miR-1246	392,48	2069,08	2846,52	1740,5	1336,07	3366,01	1540,18	1073,18	1572,87	3363,84	2863,65	2135,21	1840,46	960,14	770,53	1099,45	939,02	805,1	611,66	1331,86	2503,11	555,67	997,93	1094,61
hsa-miR-548l	2,71	0	3,52	0,83	3,38	5	1,63	0	1,1	3,84	4,45	1,82	3,2	3,96	4,14	3,88	3,75	2,07	3,72	3,25	1,33	1,29	0,45	0
hsa-miR-23a-3p	67,31	70,37	37,16	71,69	53,5	69,49	53,35	81,41	53,79	112,63	152,46	74,18	90,45	74,9	57,91	72,64	68,02	51,54	48,51	38,19	63,65	68,01	55,39	44,85
hsa-miR-1228-5p	2,99	2,59	0	2,66	12,48	5,68	4,88	4,42	9,04	3,2	10,74	2,37	19,72	2,92	4,73	7,19	5,96	2,99	1,17	5,54	0	8,58	4,95	0
hsa-miR-382-5p	17,64	6,69	9,89	7,3	9,3	38,38	8,86	6,42	2,87	17,28	12,31	12,94	16,76	17,73	15,36	15,34	17,67	6,21	24,26	0,76	31,41	10,73	23,19	0
hsa-miR-574-5p	45,6	58,5	19,35	61,57	49,06	113,54	21,88	66,81	31,08	76,58	126,26	41,56	116,08	57,37	40,38	68,36	54,77	41,19	46,75	48,32	22,77	76,59	53,81	38,68
hsa-miR-30a-3p	18,19	26,12	24,18	23,57	39,76	20,66	14,29	16,81	31,3	21,76	27,5	18,04	35,49	48,4	13,98	19,23	9,28	16,8	18,58	17,19	10,47	13,95	16,66	11,4
hsa-miR-4446-3p	24,7	11,66	9,01	14,77	5,5	49,73	13,75	0,66	9,48	30,93	20,69	0	22,92	6,88	16,15	18,26	8,17	25,31	20,73	4,97	28,58	24,89	16,66	3,55
hsa-miR-150-5p	52,11	56,77	30,12	45,97	106,16	33,83	28,76	82,52	47,4	74,23	68,89	17,5	53,97	106,61	41,95	610,22	43,73	50,85	515,62	37,24	30,91	78,52	41,66	33,07
hsa-miR-106b-3p	131,64	254,08	185,34	527,73	269,41	313,37	193,88	119,24	176,36	301,62	347,35	273,03	208,74	355,72	157,97	365,12	195,67	177,63	484,91	254,38	158,38	352,5	305,32	152,29
hsa-miR-155-5p	68,4	91,53	51,67	71,03	83,95	127,16	68,73	90,04	75,39	108,15	116,57	58,51	134,81	120,38	55,35	402,8	58,3	59,59	620,66	51,18	31,24	90,75	63,05	38,49
hsa-miR-199b-3p	154,17	210,47	118,94	178,07	125,4	388,31	128,05	163,93	150,78	368,81	365,42	190,1	250,64	246,81	174,51	151,68	116,6	173,49	129,1	94,15	108,52	202,75	164,14	42,98
hsa-miR-191-5p	305,9	421,8	692,78	660,49	479,19	1285,5	333,68	246,89	479,47	1340,84	979,7	792,48	576,68	514,91	329,33	396,98	308,96	261,62	609,9	322,37	546,92	458,7	350,36	194,71
hsa-miR-26a-5p	1033,59	1981,01	1284,42	1773,52	1386,4	4718,27	954,02	1120,53	1380,2	3978,81	3053,57	1572,02	2384,62	2741,25	1214,89	3654,74	944,98	1100,08	4031,06	977,6	1417,91	1719,37	1560,61	727,62
hsa-miR-3614-5p	3,26	13,38	12,31	12,11	15,65	14,53	8,86	7,52	19,62	20,05	23,05	6,2	2,22	8,35	0,98	5,05	0	0	2,15	15,09	7,98	0,64	5,4	3,55
hsa-miR-181b-5p	56,19	119,37	81,35	184,7	112,08	143,06	80,12	104,64	76,27	116,04	221,09	93,14	198,39	127,27	64,21	146,83	50,13	52,69	262,31	59,39	61,16	191,37	87,81	83,71
hsa-miR-493-5p	16,01	6,91	12,09	3,15	4,02	29,52	1,45	3,32	0	13,86	0	0	1,73	19,61	5,32	15,73	7,29	3,45	16,04	0,57	0	4,29	21,84	0
hsa-miR-324-5p	19,54	33,68	14,73	64,22	60,69	24,98	52,99	15,49	43,65	30,08	78,32	50,3	25,88	56,33	25,61	45,83	25,18	46,02	59,86	43,54	13,29	64,58	62,6	15,88
hsa-miR-877-5p	8,14	0,86	3,08	3,82	2,33	4,54	11,57	7,3	6,17	14,08	5,24	7,47	11,09	1,04	2,56	5,05	0	4,6	3,52	0,95	3,32	8,37	10,58	9,72
hsa-miR-574-3p	5,43	16,62	5,94	13,94	15,01	8,17	13,75	5,75	5,29	29,65	22,79	12,39	16,76	14,4	12,01	21,75	1,1	13,35	6,06	4,58	3,99	7,08	18,46	1,49
hsa-miR-1304-3p	3,26	9,28	6,6	14,44	10,36	14,31	1,63	8,19	6,61	19,62	33,01	7,47	13,8	4,38	17,14	6,21	4,64	2,53	3,33	2,86	7,15	23,6	15,54	4,11
hsa-let-7g-5p	237,5	690,13	281,86	702,97	552,15	793,64	194,42	254,19	346,98	775,37	799,21	492,47	419,7	825,78	315,54	1626,36	277,16	366,31	1822,27	360,18	106,69	680,11	592,41	136,03
hsa-miR-16-2-3p	107,48	165,14	34,3	210,09	106,58	178,26	40,87	80,31	57,76	129,9	157,69	102,07	110,9	241,39	96,71	180,04	86,13	117,58	275,22	109,05	14,46	164,56	194,99	49,52
hsa-miR-126-5p	536,61	492,61	281,86	488,39	279,56	730,29	430,98	592,89	521,79	512,79	685	350,49	717,65	789,47	460,9	380,47	388,02	529,91	441,09	279,01	283,18	684,19	596,46	267,39
hsa-miR-19a-3p	2,44	6,26	1,54	3,98	2,54	4,77	3,62	4,65	3,75	10,24	4,98	9,3	7,15	6,05	2,95	1,36	3,53	4,37	5,67	4,01	0	1,93	7,43	1,49
hsa-miR-181a-2-3p	10,04	26,77	5,94	25,06	11,42	36,79	14,47	21,24	15,87	43,3	28,55	9,66	26,62	14,81	10,05	22,14	13,25	8,28	80,59	14,51	20,77	32,61	15,31	9,34
hsa-miR-132-3p	2,44	8,42	5,72	12,11	0	7,27	4,16	2,21	11,02	13,01	17,55	8,57	23,17	8,97	4,14	6,8	1,99	6,44	9,78	5,92	4,32	4,29	12,61	0
hsa-let-7b-5p	14278,32	22210,34	14279,67	30184,72	17576,37	22040,15	21454,87	14479,67	18725,42	19391,26	26491,12	16519,04	18108,39	28509,5	13969,63	26914,54	14441,55	18007,83	29310,45	20071,45	11484,36	27788,42	25171,54	16081,93
hsa-miR-192-5p	580,04	1654,19	697,83	1196,67	638,64	775,02	2688,62	850,85	921,9	721,83	1065,36	808,7	927,62	1613,58	484,34	1189,96	1286,41	1131,83	604,62	862,25	1004,93	1097,19	1826,76	651,01
hsa-miR-323a-3p	9,23	3,89	4,4	1,16	0	12,49	3,98	3,32	8,16	3,84	6,81	2	9,86	13,14	6,11	9,13	4,86	8,97	14,47	2,67	9,14	8,8	12,83	2,8
hsa-miR-28-5p	2,99	19	7,7	7,47	10,57	22,94	1,45	15,71	3,97	21,12	14,15	3,46	17,25	8,55	7,09	18,45	3,75	3,91	26,21	0,19	0,17	4,08	5,63	0,19
hsa-miR-942-5p	2,99	0	4,4	2,49	2,11	1,36	1,81	1,33	2,2	10,67	3,14	2,55	1,97	2,92	2,17	1,75	0,22	0	6,06	0	0	1,07	4,05	1,31
hsa-miR-197-3p	9,5	41,01	24,18	24,56	34,26	38,38	4,7	8,19	16,75	78,5	57,63	23,88	37,21	21,7	12,01	16,12	16,34	10,35	18,39	21,58	5,98	12,44	6,53	6,35
hsa-miR-744-5p	65,68	85,48	68,6	100,9	79,09	226,17	34,18	21,46	55,99	134,38	132,81	77,28	89,95	56,33	27,38	51,86	52,56	58,44	74,13	63,4	78,61	59,64	53,36	25,79
hsa-miR-598-3p	13,3	8,42	6,6	36,68	27,91	26,8	3,62	10,62	1,98	16,21	19,65	11,66	17,99	12,31	6,11	16,31	7,29	12,66	31,88	18,91	6,48	24,89	9,68	13,45
hsa-miR-206	24,7	11,01	9,45	17,26	15,44	2,04	9,95	78,54	13,67	12,59	29,6	21,69	144,66	16,27	75,04	1,94	5,52	8,28	14,08	7,83	13,13	65,22	4,5	49,33
hsa-miR-210-3p	5,7	18,78	3,08	16,93	5,29	9,76	11,21	7,3	4,19	13,22	8,38	6,74	4,44	15,23	6,89	20,39	1,1	7,13	13,11	13,56	5,48	9,87	17,56	6,17
hsa-miR-200a-3p	42,89	32,81	15,39	128,78	46,31	32,02	50,28	9,07	51,36	27,09	177,86	29,16	24,15	22,95	12,41	8,74	56,31	73,4	10,95	50,23	42,05	180,65	36,25	48,96
hsa-miR-17-5p	9,77	11,44	10,11	24,23	21,78	30,43	0	13,72	6,61	9,6	29,08	26,43	3,7	19,61	9,65	6,99	14,58	14,96	27,78	18,52	0	10,94	22,07	7,85
hsa-miR-29a-3p	7,87	36,48	10,11	31,2	8,67	29,75	12,66	27,65	24,91	48,21	65,23	32,44	33,27	30,67	13	56,13	14,35	19,56	52,23	13,94	8,14	26,82	27,92	14,2
hsa-miR-30c-5p	12,49	17,05	12,31	23,23	15,44	59,95	9,22	18,36	2,42	61,01	38,77	14,76	22,67	31,71	18,12	31,27	11,48	10,81	27,19	9,36	4,65	11,16	18,24	2,8
hsa-miR-424-3p	0	6,69	0	19,58	6,13	8,63	2,89	4,87	16,75	24,96	13,62	15,13	10,35	10,43	2,17	14,37	6,4	6,21	5,67	4,97	6,98	8,37	19,81	3,55
hsa-miR-17-3p	5,97	6,69	1,1	10,29	5,71	2,04	8,32	0	2,65	5,55	14,15	2,55	5,67	4,8	4,14	6,21	0	1,84	11,15	8,02	4,82	9,65	2,93	0
hsa-miR-151a-5p	14,39	15,54	10,11	29,37	16,71	59,49	9,95	10,62	10,8	37,76	54,49	5,1	32,78	25,45	12,8	26,22	9,05	12,89	38,34	7,83	22,6	29,18	31,07	5,05
hsa-miR-21-5p	1160,34	1986,84	919,23	3971,88	1605,48	3553,12	1246,1	2044,6	1437,08	3134,54	5008,51	1513,15	3164,13	1697,03	1015,56	3346,13	837,66	895,76	3423,9	1251,27	997,12	2223,98	1589,88	1072,38
hsa-miR-223-3p	29,59	221,26	69,92	216,73	250,38	431,91	15,37	104,86	78,7	966,49	542,24	252,62	192,23	127,48	93,17	115,36	25,18	25,31	57,7	54,24	41,38	31,75	79,03	6,35
hsa-miR-215-5p	21,99	6,48	12,97	50,78	37,64	30,2	19,89	23,67	24,91	28,58	44,01	19,5	11,09	10,64	16,74	7,96	52,56	53,38	3,72	10,5	9,47	30,25	16,21	0,75
hsa-miR-186-5p	79,26	115,49	49,91	194,99	113,98	185,52	60,04	95,35	98,76	204,56	195,68	123,39	160,68	160,65	70,51	141,97	60,95	89,97	165,29	76,96	48,86	133,45	135,1	47,28
hsa-miR-454-5p	3,26	8,2	0	10,79	14,59	5,9	0	6,42	8,16	8,32	10,22	4,74	4,68	11,47	4,33	14,95	2,87	0,23	10,37	8,78	9,31	7,08	9,01	3,18
hsa-miR-301a-3p	2,44	4,96	2,42	6,31	4,65	9,54	4,52	6,42	0	9,81	1,83	4,01	6,9	4,8	4,14	3,88	0	0	8,41	0	0	2,57	4,5	0,75
hsa-miR-140-5p	1,09	3,02	0	2,99	3,17	4,77	1,27	0,88	4,63	8,96	6,29	6,56	5,18	4,17	2,17	4,66	4,2	5,52	1,76	0	0	1,93	1,35	0
hsa-miR-582-3p	0,27	3,67	3,3	4,81	3,81	8,86	3,07	1,55	2,2	2,56	13,36	7,66	5,42	1,46	1,58	5,05	1,99	1,61	0,2	0	9,81	0	1,8	2,06
hsa-miR-221-3p	863,68	1273,83	646,39	1499,54	729,57	1721,72	821,81	1310,12	710,71	1783,67	2244,92	1019,21	2025,79	1282,69	815,64	1476,81	770,96	872,52	1195,74	664,59	546,09	1344,13	1227,59	697,91
hsa-miR-7847-3p	4,61	9,71	18,91	10,12	3,59	15,44	18,45	6,42	3,75	19,41	10,22	14,03	9,36	12,73	7,29	3,69	3,31	11,5	4,5	3,25	4,15	0,43	1,8	2,06
hsa-miR-181d-5p	8,14	6,04	26,6	18,25	10,78	29,75	0	11,73	10,8	32,42	29,86	7,66	32,28	16,48	1,18	8,74	10,38	9,89	18,39	5,92	18,78	3,86	14,41	5,98
hsa-miR-320d	5,7	6,69	3,08	3,48	3,59	5	2,17	5,97	2,87	10,88	8,64	6,01	3,45	4,8	5,71	5,24	3,53	5,52	2,93	1,15	4,99	6,65	12,61	2,24
hsa-miR-320c	9,5	20,08	8,13	15,77	12,05	15,67	14,47	26,99	5,95	18,56	20,43	18,59	25,63	22,12	12,01	20,39	11,48	20,71	11,15	7,83	11,47	20,81	19,36	10,09
hsa-miR-139-3p	14,93	11,44	13,41	4,31	4,65	15,9	10,13	13,05	17,19	10,03	8,64	6,01	8,38	12,94	7,09	0,78	7,95	20,25	7,24	5,73	35,73	14,37	12,61	3,18
hsa-miR-320b	176,15	213,49	227,12	240,13	131,75	235,03	331,51	281,4	218,24	224,19	306,22	219,99	425,37	234,92	192,04	254,81	185,07	198,57	172,52	149,15	186,3	317,96	285,28	204,98
hsa-miR-151a-3p	675,58	505,78	492,93	709,44	371,98	1411,76	621,79	493,12	662,21	783,9	1011,66	289,98	645,94	608,38	413,63	608,67	533,11	642,65	586,62	447,45	855,37	938,42	598,71	554,97
hsa-let-7i-5p	1452,12	2951,33	1814,94	4102,81	2740,02	4135,13	1981,11	1468,3	2157,27	3188,08	3799,6	2070,14	2620,22	3742,9	1401,81	4430,24	1519,4	1974,89	6643,77	2311,18	1710,23	3901,3	2850,35	1483,47
hsa-miR-652-3p	8,41	14,46	7,48	13,94	4,44	11,81	6,33	13,94	4,41	13,86	11	6,93	10,6	16,06	8,08	9,71	9,5	12,19	15,26	18,33	11,8	15,23	16,21	14,01
hsa-miR-148b-3p	20,63	57,64	21,99	103,72	65,56	249,33	28,58	39,38	22,04	135,24	106,35	79,83	53,73	47,78	30,14	68,56	20,76	22,32	68,85	21,77	29,58	31,11	36,03	10,09
hsa-miR-1843	21,17	2,16	12,53	0,5	13,32	43,15	17	6,86	7,94	43,3	3,67	18,04	15,28	16,9	12,21	14,57	13,69	10,58	25,82	25,02	17,78	2,57	18,91	8,6
hsa-miR-204-5p	1,36	8,42	1,76	3,15	2,96	3,41	2,35	2,65	1,54	1,71	0	5,47	5,91	6,88	0,79	0,19	3,53	2,53	1,56	1,34	0	0	1,58	0
hsa-miR-454-3p	1,63	9,71	2,64	3,98	9,73	6,59	2,17	4,2	2,42	18,77	10,22	4,01	0,99	10,64	2,56	7,19	4,64	4,6	18,19	8,02	0	6,65	1,58	0
hsa-miR-193a-5p	1,9	1,73	0	0	0	4,54	6,51	1,77	1,98	2,56	4,72	1,82	12,57	2,29	1,77	4,08	0	0	2,35	0	5,15	0	5,4	0
hsa-miR-24-3p	64,87	96,06	49,69	81,65	98,55	153,73	57,15	135,83	76,94	229,94	201,96	129,22	136,53	132,48	97,5	95,94	53,44	58,21	84,11	43,54	44,7	89,25	102,22	26,72
hsa-miR-140-3p	202,21	273,94	218,32	405,92	277,66	515,93	233,12	209,06	246,02	552,46	385,07	381,29	361,54	325,26	211,34	304,72	139,35	205,47	425,25	183,72	226,18	271,83	239,8	166,49
hsa-miR-27b-3p	70,84	212,63	79,37	185,7	70,42	179,62	237,1	147,56	93,91	158,06	281,86	124,67	215,64	203,63	92,18	171,88	103,13	109,06	106,21	60,54	101,21	199,53	206,93	55,12
hsa-miR-629-5p	44,24	57,2	24,18	56,59	53,08	51,09	24,23	24,11	43,87	56,1	76,75	63,43	50,03	72,19	46,29	25,44	28,27	40,73	51,84	62,26	46,03	34,54	45,71	31,39
hsa-miR-28-3p	206,55	284,51	335,07	323,11	261,59	629,69	334,59	230,52	324,71	582,54	552,46	266,83	356,85	231,79	160,72	401,83	221,06	178,55	415,47	158,13	400,01	249,09	228,09	228,15
hsa-let-7d-5p	182,13	482,46	201,83	538,01	378,32	843,37	199,3	252,42	322,73	647,39	701,77	340,28	364	581,88	269,84	675,48	205,16	279,79	891,77	321,98	133,28	560,39	541,97	132,3
hsa-miR-2110	0	1,73	1,32	1,66	1,69	3,63	0,9	1,99	4,19	4,27	4,19	6,01	1,73	5,63	1,18	5,44	3,09	1,61	5,09	3,63	0	5,36	3,15	2,99
hsa-miR-128-3p	14,11	46,63	13,85	72,52	48,64	84,25	4,52	41,15	21,38	123,72	82,25	40,46	64,08	60,92	18,32	70,31	24,07	16,57	77,26	24,44	10,97	37,97	49,99	2,62
hsa-miR-30a-5p	786,59	989,32	553,83	830,75	739,51	723,48	1511,96	986,68	1116,99	746,79	1238,77	608,76	1148,69	1010	763,84	853,58	545,92	846,75	402,56	553,64	1146,86	1136,88	1040,03	822,18
hsa-miR-432-5p	141,41	33,03	90,58	12,61	5,71	216,63	56,07	17,03	23,37	93,64	27,5	18,77	25,14	42,56	104,59	79,63	95,62	40,04	109,34	10,12	73,79	7,94	58,54	10,65
hsa-miR-99b-5p	58,08	79,65	83,55	71,36	39,12	187,11	107,07	162,6	78,04	156,14	143,29	63,25	137,76	101,61	102,42	72,44	63,38	101,24	60,64	62,83	102,37	91,18	74,53	86,52

hsa-let-7d-3p	101,51	141,18	66,18	178,73	155,01	193,02	90,25	109,73	117,94	220,99	300,72	148,73	137,76	162,11	97,89	175,57	96,95	56,6	169,4	96,25	81,93	140,53	141,63	49,33
hsa-miR-378a-3p	57,27	126,71	69,92	113,68	83,53	115,81	196,41	124,11	59,52	137,37	96,14	105,17	161,92	131,23	64,21	67,59	53,22	63,51	96,63	93,39	83,59	68,01	131,05	92,68
hsa-miR-200c-3p	5,43	5,4	2,64	14,6	1,9	3,86	3,07	5,09	4,63	13,44	14,93	12,58	5,67	0,83	3,35	8,55	11,7	6,9	13,11	3,63	0	14,8	3,15	0
hsa-miR-29c-3p	3,8	15,97	5,94	12,45	8,88	10,67	8,14	17,48	2,87	7,89	22	7,84	9,61	25,87	7,29	16,7	2,87	10,35	13,69	4,39	4,49	16,09	18,24	0
hsa-miR-125b-2-3p	4,61	15,76	8,13	17,42	2,33	3,18	20,8	12,17	5,07	4,27	14,93	8,02	15,53	8,35	3,35	25,64	5,3	8,28	5,67	11,46	6,15	5,36	13,28	13,83
hsa-miR-30e-5p	249,71	392,01	173,47	469,31	329,47	616,98	361,89	338,04	283,49	479,94	610,61	403,35	483,28	501,56	276,15	376,58	212,89	289,23	459,28	226,5	281,02	388,76	443,8	309,81
hsa-miR-345-5p	14,66	57,2	30,34	55,59	29,39	55,63	7,05	31,41	24,69	97,27	81,47	50,85	35,49	57,79	32,5	43,5	23,85	20,94	30,32	11,46	31,08	14,37	24,99	7,29
hsa-miR-125a-5p	16,01	5,83	15,39	13,61	13,75	25,66	16,82	34,51	23,81	17,49	33,53	30,07	19,72	19,82	13,98	25,25	13,91	14,96	16,63	7,45	16,45	21,67	11,48	2,8
hsa-miR-10399-3p	2,17	1,94	14,73	4,48	5,92	1,36	6,87	2,65	1,76	4,05	3,93	4,74	5,67	1,25	1,97	3,69	4,86	0	6,06	1,53	14,13	2,57	3,83	7,1
hsa-miR-671-3p	7,06	9,07	9,45	6,8	8,67	37,92	6,51	1,77	11,68	28,16	9,69	2,37	9,61	6,05	11,42	9,71	7,07	0	10,56	8,21	9,47	7,94	4,95	9,72
hsa-miR-103a-3p	961,93	1989,43	1039,94	2181,93	1105,99	1788,03	1893,39	1356,13	1091,86	1138,84	1901,5	996,98	1926,23	2735,62	931,06	1904,28	923,56	1251,48	2053,87	1091,61	1742,47	2455,69	2104,38	2120,28
hsa-miR-25-3p	2055,5	3849,12	2093,5	6099,71	4009,48	4661,5	1842,57	2214,28	2549,44	4487,97	5176,68	3294,22	3486,72	4609,99	2061,45	4823,53	2161,61	1986,17	7435,98	3429,91	1451,48	3824,71	3914,93	1732,36
hsa-miR-584-5p	95,27	59,58	105,53	85,13	37,01	211,87	68,91	46,02	84,87	144,84	105,83	51,22	88,97	85,33	71,89	71,86	112,41	90,2	93,5	64,36	123,64	115,43	65,97	51,76
hsa-miR-223-5p	11,67	46,63	11,21	27,05	36,8	77,21	4,16	29,2	36,37	83,4	62,61	45,57	40,42	26,5	19,5	24,08	5,08	18,64	14,08	23,49	11,3	9,65	18,91	2,43
hsa-miR-1180-3p	10,31	1,3	8,13	16,93	9,94	8,17	6,51	4,2	9,7	12,37	14,15	9,11	6,41	13,35	7,48	3,5	10,16	8,97	18,97	12,99	4,99	21,45	13,74	5,23
hsa-miR-660-5p	25,79	32,6	26,16	46,47	15,23	33,38	25,86	25,66	28,66	33,28	64,96	42,65	57,18	49,24	16,35	33,6	22,75	38,43	43,82	24,25	4,15	37,55	27,02	31,58
hsa-miR-371b-5p	0	4,96	2,42	3,98	3,81	7,27	7,6	1,55	7,05	10,03	12,84	8,38	10,35	5,22	8,08	2,72	1,1	0	0	0	5,15	1,07	4,5	1,31
hsa-miR-194-5p	0	7,12	9,01	9,96	0	6,13	8,14	1,55	3,31	2,99	11	9,11	10,6	8,55	3,94	10,1	1,99	0	4,89	10,31	0	6,22	7,66	3,55
hsa-miR-30e-3p	35,56	99,95	52,33	84,3	81,42	144,65	16,28	34,73	40,34	194,32	182,84	70,54	98,09	61,34	38,41	95,55	30,26	37,74	116,97	35,52	65,81	35,83	61,02	16,63
hsa-miR-340-5p	39,63	36,91	41,77	52,77	45,68	84,7	20,26	30,53	26,89	75,08	90,9	45,2	35,98	47,57	19,7	26,02	29,15	20,02	39,9	34,18	38,39	37,12	44,13	19,43
hsa-miR-532-5p	87,67	104,48	52,33	159,31	123,5	133,3	121,72	76,32	112,65	85,54	186,77	82,2	85,76	142,91	117,79	126,24	68,68	66,5	146,7	96,44	50,69	176,36	114,83	69,7
hsa-miR-10b-5p	3099,68	2790,94	3564,14	2911,95	2059,93	3231,8	6371,59	5159,73	4248,62	3324,59	4577,86	3317,92	6930,32	3153,72	3638,75	2824,67	2642,38	3767,79	1540,79	2261,14	5452,6	3756,27	2590,06	7080,8
hsa-miR-142-5p	402,8	926,29	296,59	1230,86	706,52	914,45	496,45	653,07	539,21	1157,19	1542,11	749,83	854,18	1076,76	500,69	1284,35	424,24	456,05	1242,1	559,56	285,51	931,34	897,05	327,38
hsa-miR-320a-3p	1529,75	1736	1335,21	2158,69	1367,16	2274,66	1611,25	2610,28	1526,58	1856,41	2358,61	1741,89	3388,15	2052,96	1392,75	2363,99	1696,95	1767,12	2489,68	1516,72	884,12	2069,72	2007,34	1375,46
hsa-miR-409-3p	83,33	31,08	31,88	14,11	15,23	158,73	42,86	13,72	19,4	95,77	12,57	10,02	55,45	30,04	32,7	44,67	41,3	36,82	127,93	12,8	36,73	12,87	50,21	14,57
hsa-miR-101-3p	393,84	552,4	406,96	968,16	566,11	638,09	689,43	361,27	447,06	482,07	762,02	453,11	526,9	838,92	333,86	612,36	443,45	435,11	842,09	468,27	603,59	959,45	648,47	646,34
hsa-miR-183-5p	29,86	62,82	76,95	55,59	91,99	79,25	89,16	38,05	60,4	44,15	48,46	68,9	37,46	88,67	35,85	68,56	76,85	86,75	78,83	100,07	73,45	72,09	104,25	94,18
hsa-miR-27a-3p	8,69	31,95	5,94	19,91	17,55	36,56	13,75	15,71	14,11	40,95	39,03	24,97	28,83	42,35	4,73	16,12	7,29	13,35	9,19	9,36	5,65	21,88	23,42	5,23
hsa-miR-4488	7,33	8,2	0	8,3	17,76	3,63	2,89	15,49	39,68	13,86	3,93	4,19	15,28	20,24	3,94	8,55	12,15	18,87	2,54	14,51	9,31	10,94	12,61	2,99
hsa-miR-199a-3p	309,15	420,72	237,67	356,3	250,59	778,66	255,91	328,08	301,35	740,6	731,11	380,02	501,03	493,84	349,02	303,56	233,43	346,75	258	188,3	217,04	405,49	328,29	86,14
hsa-miR-374b-5p	4,89	6,91	8,35	9,13	8,25	17,26	2,53	2,65	1,76	17,49	8,91	4,92	7,15	8,97	0	2,14	4,2	1,84	9,19	6,3	6,32	6,22	2,03	4,86
hsa-miR-142-3p	13,3	33,89	5,28	32,19	12,69	27,25	8,86	15,71	17,19	23,89	41,39	25,33	15,53	22,12	6,11	23,31	8,39	14,73	27,78	15,28	8,64	9,01	22,07	7,1
hsa-miR-3158-3p	2,17	2,59	3,74	6,97	1,69	4,54	7,23	4,87	6,39	0	3,14	2,19	4,44	13,56	5,32	5,44	7,51	3,45	10,76	8,4	4,15	9,23	6,75	10,84
hsa-miR-181a-5p	632,42	866,49	1032,24	1362,46	838,9	1561,63	708,42	748,42	907,57	1389,48	1661,82	863,56	1240,36	1120,99	634,43	1567,51	572,2	553,61	2252,8	662,3	1224,97	1305,3	681,35	765,74
hsa-miR-328-3p	30,13	48,14	58,04	79,66	41,45	88,79	21,34	41,59	50,04	140,78	123,38	51,4	73,44	75,53	45,11	74,77	20,1	31,06	69,05	19,48	0	38,4	41,66	5,98
hsa-miR-381-3p	9,77	2,37	9,23	0	0	6,36	13,38	7,3	2,87	6,19	2,88	3,1	3,7	6,47	7,68	6,6	0,66	3,45	9,58	0	4,82	1,29	9,01	6,35
hsa-let-7f-5p	444,32	1440,7	835,69	1570,56	1252,75	2807,39	550,89	695,77	854,88	1895,44	1810,08	879,78	1335,99	1496,74	593,07	2107,04	543,27	822,13	3184,08	927,56	749,01	1435,53	1266,1	456,12
hsa-miR-486-5p	123775,6	136611,62	206372,59	215087,66	160656,68	149362,98	225160,12	108309,15	142619,09	78055,01	113814,39	143669,7	125526,5	160076,98	99785,27	120038,6	201234,02	164798	151652,31	215982,57	213610,46	176473,92	190386,08	308624,73
hsa-miR-375-3p	58,08	75,77	75,85	175,08	37,01	50,41	97,3	52,43	65,69	36,26	252,26	79,83	49,54	44,44	39,2	68,75	92,53	121,26	18,39	82,5	25,59	165,63	44,36	31,02
hsa-miR-4466	3,26	0	3,74	8,46	0	5,45	6,51	5,53	11,02	4,69	5,5	3,46	5,67	2,71	1,38	0,97	8,17	2,99	0,2	8,98	0	2,57	5,18	8,78
hsa-miR-885-3p	8,14	19,43	13,63	20,41	10,57	8,86	107,07	32,74	12,57	1,92	25,67	10,21	29,57	28,58	3,94	19,03	30,92	8,05	0,39	21,58	20,27	15,02	36,7	24,1
hsa-let-7a-5p	1341,65	3916,26	2006,22	4070,62	3326,01	6080,97	1707,83	2099,46	2274,32	4815,39	4581,79	2389,65	3519,5	3796,52	1660,43	5447,35	1607,29	2389,52	7063,35	2564,03	1613,34	3649,86	3254,07	1287,08
hsa-miR-7706	4,07	8,2	4,18	5,97	2,33	7,49	0	2,88	6,61	4,69	1,05	4,74	1,48	13,77	4,92	5,44	10,38	1,38	9	4,2	4,99	6,01	13,06	6,17
hsa-miR-7704	55,1	29,14	24,62	32,86	55,62	34,52	62,03	28,32	100,52	57,59	108,19	63,97	60,38	51,95	46,48	86,23	11,04	6,67	173,31	57,29	21,27	74,02	27,7	11,4
hsa-miR-486-3p	139,24	176,8	101,58	254,24	172,35	161,45	152,64	122,78	124,33	80,63	165,82	199,58	104	239,93	154,42	158,09	151,72	168,2	285,98	152,02	16,29	223,99	221,56	79,79
hsa-miR-370-3p	15,47	11,44	15,61	4,15	4,02	40,65	18,99	3,76	3,97	21,97	7,6	2,92	13,31	11,89	9,06	25,05	12,81	12,89	14,47	6,88	24,1	3,86	20,26	1,68
hsa-miR-4732-5p	14,39	6,91	4,84	9,13	13,11	4,54	12,84	4,65	3,53	7,89	4,72	4,37	10,35	10,43	5,91	5,05	5,52	12,43	8,22	10,5	4,49	13,52	15,54	1,31
hsa-miR-4448	16,29	5,83	36,5	26,55	23,05	17,26	51	12,17	31,96	16,64	26,72	9,11	28,83	17,73	14,77	57,68	23,63	10,81	258,79	9,36	44,04	41,19	8,33	45,59
hsa-miR-4443	6,24	11,66	16,05	37,67	37,01	27,02	22,97	7,96	29,76	21,33	34,84	37,73	16,76	7,09	13,39	25,64	12,59	0,92	2,54	13,37	19,78	11,16	21,62	14,57
hsa-miR-92a-3p	13746,6	18965,42	10471,48	24374,26	16737,04	21989,29	15572,32	14437,64	13384,28	14746,94	21893,36	14886,51	17451,36	23467,01	12207,97	23579,67	12298,71	15110,26	28735,17	16419,25	8585,23	23518,53	25677,7	10683,79
hsa-let-7a-3p	2,17	3,67	5,06	6,8	4,44	8,17	0	6,86	4,41	20,9	7,86	5,29	9,36	10,85	8,27	8,16	4,86	6,21	5,67	4,2	0	14,37	15,31	2,24
hsa-miR-423-5p	1508,58	1947,34	1263,97	2434,51	1572,92	2104,58	2085,82	2157,87	1723,43	2048,6	2728,48	1866,74	2557,87	2333,58	1279,89	1855,34	1579,69	1728,46	1676,35	1733,67	1446,33	2229,78	2452,71	1619,31
hsa-miR-24-2-5p	2,17	0,43	7,7	1,83	0,63	6,81	1,09	3,76	3,53	7,04	1,83	4,01	4,44	2,71	1,38	5,63	0	0	1,56	0	7,31	3	6,08	0
hsa-miR-4732-3p	20,9	11,44	36,94	40,66	24,32	13,4	23,33	5,75	22,04	10,88	24,36	32,99	19,47	50,28	9,45	30,3	19,65	35,43	51,25	37,81	12,63	21,24	22,74	20,93
hsa-miR-16-5p	1207,3	1788,46	590,54	2588,34	1612,46	1753,06	1073,93	1357,68	1228,98	1202,41	2091,42	1594,44	1477,69	3038,14	1114,24	1587,13	1009,25	1424,51	2329,48	1583,18	488,76	2335,76	2779,87	762,57
hsa-miR-92b-3p	311,05	220,83	266,91	311,32	204,28	260,23	346,34	272,55	339,04	181,31	411,26	282,87	307,57	350,51	254,68	231,7	368,37	316,61	283,24	316,83	163,03	356,36	312,75	278,79
hsa-miR-342-5p	5,7	14,46	16,93	32,03	9,94	16,58	26,95	17,03	11,68	19,62	31,96	16,22	5,67	14,4	12,41	134,98	2,43	16,8	120,3	15,85	11,8	22,74	21,84	11,96
hsa-miR-423-3p	95,27	306,32	143,13	225,53	214,64	421,01	127,32	175,21	172,17	447,73	439,03	216,89	271,09	271,02	135,32	236,36	148,85	101,7	273,85	131,77	143,25	157,05	183,51	69,88
hsa-miR-22-3p	685,62	941,83	374,64	1186,05	723,65	933,3	849,12	864,34	635,98	762,36	1421,09	687,5	1220,16	1358,63	515,85	876,49	565,8	649,78	827,61	728	469,31	1299,93	1345,81	569,92
hsa-miR-144-5p	34,2	115,06	47,71	85,96	103,83	103,55	41,42	36,5	80,9	84,04	88,8	85,85	59,89	143,54	30,53	116,14	54,33	74,78	152,96	60,16	37,39	110,92	96,82	51,57
hsa-miR-15a-5p	73,56	125,2	39,35	226,69	116,31	123,76	57,69	117,25	63,93	86,39	163,72	108,26	134,07	212,39	78,2	145,27	86,57	91,81	218,1	97,59	57	192,23	227,64	83,9
hsa-miR-23b-3p	29,04	23,31	15,61	29,71	10,15	21,12	27,67	35,18	26,23	31,36	63,39	26,25	35	35,05	28,56	32,63	28,05	26,92	18,97	9,93	31,58	29,18	30,17	15,51
hsa-miR-941	96,08	236,81	182,04	149,36	198,15	340,85	155,18	107,96	204,13	593,42	249,64	291,44	185,33	195,07	155,8	152,65	86,57	60,74	119,12	127,57	122,31	72,52	132,62	99,78
hsa-miR-184	3,26	2,37	2,2	2,66	2,75	3,86	5,61	1,11	12,12	0,85	1,05	12,94	0,99	1,04	2,17	2,14	2,43	1,15	2,74	2,1	0	0	3,6	0
TPM>1.0	207	208	203	208	199	212	204	206	209	211	211	210	210	212	209	208	199	197	207	198	192	206	208	189
TPM>5.0	168	183	170	182	170	187	173	175	166	190	190	186	190	195	169	188	166	166	183	167	170	183	184	154
TPM>10.0	141	152	145	160	150	164	143	151	145	176	173	150	169	166	135	155	142	149	163	138	144	152	165	128

Supplement Table 2		mir-486-5p					
Parameter	Timepoint	Baseline		1 hour after exposure to weightlessness		24 hours after exposure to weightlessness	
		r	p-value	r	p-value	r	p-value
age (years)	Baseline	0,036	0,933	0,539	0,168	0,407	0,317
weight (kg)	Baseline	0,238	0,570	-0,381	0,352	-0,095	0,823
height (cm)	Baseline	0,143	0,736	-0,643	0,086	-0,286	0,493
Creatinin (µmol/l)	Baseline	-0,214	0,610	-0,619	0,102	-0,095	0,823
	1 hour after exposure to weightlessness	-0,651	0,081	-0,470	0,240	0,120	0,776
	24 hours after exposure to weightlessness	-0,238	0,570	-0,690	0,058	0,119	0,779
GFR (ml/min/1,73 m²)	Baseline	0,262	0,531	-0,071	0,867	-0,310	0,456
	1 hour after exposure to weightlessness	0,452	0,260	-0,095	0,823	-0,238	0,570
	24 hours after exposure to weightlessness	0,119	0,779	-0,167	0,693	-0,476	0,233
CRP (mg/l)	Baseline	0,393	0,336	-0,160	0,706	-0,393	0,336
	1 hour after exposure to weightlessness	0,123	0,772	-0,049	0,908	-0,196	0,641
	24 hours after exposure to weightlessness	0,140	0,740	0,077	0,857	-0,077	0,857
BNP (pg/ml)	Baseline	0,114	0,788	0,355	0,388	0,038	0,929
	1 hour after exposure to weightlessness	0,243	0,563	-0,115	0,786	-0,383	0,349
	24 hours after exposure to weightlessness	-0,171	0,685	-0,016	0,971	-0,203	0,630
CK (U/l)	Baseline	-0,071	0,867	-0,667	0,071	0,024	0,955
	1 hour after exposure to weightlessness	-0,071	0,867	-0,667	0,071	0,024	0,955
	24 hours after exposure to weightlessness	-0,143	0,736	-,833*	0,010	0,048	0,911
Myoglobin (µg/l)	Baseline	-0,323	0,435	-0,635	0,091	0,132	0,756
	1 hour after exposure to weightlessness	-0,405	0,320	-,714*	0,047	0,119	0,779
	24 hours after exposure to weightlessness	0,048	0,911	-,714*	0,047	0,381	0,352

*** p<0.05 ** p<0.01**

Supplement Table 3		mir-24-3p					
**Parameter**	**Timepoint**	**Baseline**		**1 hour after exposure to weightlessness**		**24 hours after exposure to weightlessness**	
		**r**	**p-value**	**r**	**p-value**	**r**	**p-value**
**age (years)**	Baseline	0,455	0,257	-0,707	0,050	-0,132	0,756
**weight (kg)**	Baseline	0,286	0,493	-0,310	0,456	-0,238	0,570
**height (cm)**	Baseline	0,119	0,779	-0,024	0,955	0,024	0,955
**Creatinin (µmol/l)**	Baseline	0,262	0,531	0,024	0,955	0,143	0,736
	1 hour after exposure to weightlessness	0,627	0,096	-0,410	0,313	-0,048	0,910
	24 hours after exposure to weightlessness	0,571	0,139	-0,310	0,456	-0,357	0,385
**GFR (ml/min/1,73 m²)**	Baseline	-0,381	0,352	0,238	0,570	0,048	0,911
	1 hour after exposure to weightlessness	-0,667	0,071	0,524	0,183	-0,024	0,955
	24 hours after exposure to weightlessness	-0,548	0,160	0,429	0,289	0,333	0,420
**CRP (mg/l)**	Baseline	0,282	0,498	-0,049	0,908	0,160	0,706
	1 hour after exposure to weightlessness	0,417	0,304	-0,123	0,772	0,196	0,641
	24 hours after exposure to weightlessness	0,319	0,441	-0,089	0,833	0,128	0,763
**BNP (pg/ml)**	Baseline	-0,076	0,858	0,127	0,765	-0,076	0,858
	1 hour after exposure to weightlessness	0,128	0,763	-0,077	0,857	-0,243	0,563
	24 hours after exposure to weightlessness	0,514	0,192	-0,234	0,577	-0,078	0,854
**CK (U/l)**	Baseline	0,619	0,102	-0,452	0,260	-0,667	0,071
	1 hour after exposure to weightlessness	0,619	0,102	-0,452	0,260	-0,667	0,071
	24 hours after exposure to weightlessness	0,500	0,207	-0,238	0,570	-0,595	0,120
**Myoglobin (µg/l)**	Baseline	0,743*	0,035	-0,192	0,649	-0,311	0,453
	1 hour after exposure to weightlessness	0,619	0,102	-0,048	0,911	-0,238	0,570
	24 hours after exposure to weightlessness	0,310	0,456	0,071	0,867	-0,643	0,086

*** p<0.05 ** p<0.01**

Supplement Table 4		mir-223-3p					
**Parameter**	**Timepoint**	**Baseline**		**1 hour after exposure to weightlessness**		**24 hours after exposure to weightlessness**	
		**r**	**p-value**	**r**	**p-value**	**r**	**p-value**
**age (years)**	Baseline	-0,455	0,257	-,755*	0,031	-0,180	0,670
**weight (kg)**	Baseline	-0,429	0,289	0,143	0,736	-0,381	0,352
**height (cm)**	Baseline	-0,429	0,289	0,238	0,570	-0,476	0,233
**Creatinin (µmol/l)**	Baseline	-0,238	0,570	0,071	0,867	-0,619	0,102
	1 hour after exposure to weightlessness	0,096	0,820	-0,217	0,606	-,952**	0,000
	24 hours after exposure to weightlessness	-0,024	0,955	0,167	0,693	-,738*	0,037
**GFR (ml/min/1,73 m²)**	Baseline	-0,119	0,779	0,476	0,233	0,214	0,610
	1 hour after exposure to weightlessness	-0,143	0,736	,738*	0,037	0,524	0,183
	24 hours after exposure to weightlessness	-0,143	0,736	0,500	0,207	0,119	0,779
**CRP (mg/l)**	Baseline	-0,123	0,772	-0,221	0,599	0,037	0,931
	1 hour after exposure to weightlessness	-0,025	0,954	-0,393	0,336	-0,086	0,840
	24 hours after exposure to weightlessness	0,026	0,952	-0,396	0,332	0,064	0,881
**BNP (pg/ml)**	Baseline	0,495	0,213	-0,101	0,811	0,545	0,162
	1 hour after exposure to weightlessness	0,587	0,126	0,064	0,881	0,230	0,584
	24 hours after exposure to weightlessness	,733*	0,039	-0,312	0,452	-0,062	0,883
**CK (U/l)**	Baseline	0,405	0,320	0,095	0,823	-0,524	0,183
	1 hour after exposure to weightlessness	0,405	0,320	0,095	0,823	-0,524	0,183
	24 hours after exposure to weightlessness	0,333	0,420	0,333	0,420	-0,571	0,139
**Myoglobin (µg/l)**	Baseline	0,443	0,272	-0,024	0,955	-0,551	0,157
	1 hour after exposure to weightlessness	0,452	0,260	0,095	0,823	-0,571	0,139
	24 hours after exposure to weightlessness	0,095	0,823	0,571	0,139	-0,214	0,610

*** p<0.05 ** p<0.01**

Supplement Table 5		mir-941					
Parameter	Timepoint	Baseline		1 hour after exposure to weightlessness		24 hours after exposure to weightlessness	
		r	p-value	r	p-value	r	p-value
age (years)	Baseline	-0,347	0,399	-0,443	0,272	0,299	0,471
weight (kg)	Baseline	0,262	0,531	-0,095	0,823	-0,214	0,610
height (cm)	Baseline	0,286	0,493	-0,167	0,693	-0,310	0,456
Creatinin (µmol/l)	Baseline	0,071	0,867	-0,214	0,610	-0,262	0,531
	1 hour after exposure to weightlessness	0,048	0,910	-0,265	0,526	-0,410	0,313
	24 hours after exposure to weightlessness	0,143	0,736	-0,214	0,610	-0,214	0,610
GFR (ml/min/1,73 m²)	Baseline	0,310	0,456	0,571	0,139	-0,333	0,420
	1 hour after exposure to weightlessness	0,143	0,736	0,690	0,058	-0,048	0,911
	24 hours after exposure to weightlessness	0,333	0,420	0,643	0,086	-0,524	0,183
CRP (mg/l)	Baseline	0,442	0,273	-0,589	0,124	-0,049	0,908
	1 hour after exposure to weightlessness	0,258	0,538	-0,577	0,134	-0,025	0,954
	24 hours after exposure to weightlessness	0,102	0,810	-0,562	0,147	0,166	0,694
BNP (pg/ml)	Baseline	0,000	1,000	-0,114	0,788	0,330	0,425
	1 hour after exposure to weightlessness	0,600	0,116	-0,358	0,385	-0,077	0,857
	24 hours after exposure to weightlessness	0,483	0,225	-0,483	0,225	-0,171	0,685
CK (U/l)	Baseline	0,333	0,420	-0,667	0,071	0,024	0,955
	1 hour after exposure to weightlessness	0,333	0,420	-0,667	0,071	0,024	0,955
	24 hours after exposure to weightlessness	0,262	0,531	-0,405	0,320	-0,048	0,911
Myoglobin (µg/l)	Baseline	0,168	0,691	-0,563	0,146	0,000	1,000
	1 hour after exposure to weightlessness	0,119	0,779	-0,429	0,289	-0,048	0,911
	24 hours after exposure to weightlessness	-0,143	0,736	-0,071	0,867	0,357	0,385

*** p<0.05** **** p<0.01**

Supplement Table 6

Hypergeometric Test

Name	Hits	Pval
p53 signaling pathway	23	1,19E-08
Cell cycle	28	0,0000016
HTLV-I infection	33	0,000133
Pathways in cancer	42	0,000691
Bladder cancer	10	0,000691
Prostate cancer	18	0,000804
Chronic myeloid leukemia	16	0,000933
Small cell lung cancer	16	0,00266
Glioma	14	0,0028
Melanoma	13	0,0145
Non-small cell lung cancer	11	0,0145
Pancreatic cancer	13	0,0145
Legionellosis	9	0,0234

Empirical sampling

Name	Hits	Pval
Thiamine metabolism	1	< 0.001
Vitamin B6 metabolism	2	< 0.001
Lipoic acid metabolism	1	< 0.001
Sulfur relay system	2	< 0.001
Cytosolic DNA-sensing pathway	3	< 0.001
Asthma	1	< 0.001
Glycosaminoglycan degradation	4	0,001
Lysosome	3	0,001
Maturity onset diabetes of the young	3	0,001
p53 signaling pathway	23	0,003
NOD-like receptor signaling pathway	9	0,004
Aldosterone-regulated sodium reabsorption	6	0,005
Legionellosis	9	0,005
Propanoate metabolism	3	0,007
Amyotrophic lateral sclerosis (ALS)	6	0,007
Bladder cancer	10	0,008
Toll-like receptor signaling pathway	13	0,01
Intestinal immune network for IgA production	3	0,01
Small cell lung cancer	16	0,01
Glycerophospholipid metabolism	8	0,011
Nicotinate and nicotinamide metabolism	3	0,011
Tight junction	14	0,011
HTLV-I infection	33	0,011
Steroid biosynthesis	3	0,012
Cytokine-cytokine receptor interaction	20	0,012
Apoptosis	12	0,014
Leishmaniasis	7	0,014
Non-small cell lung cancer	11	0,014
Staphylococcus aureus infection	3	0,015
MAPK signaling pathway	30	0,016
African trypanosomiasis	3	0,016
Prostate cancer	18	0,016
Melanoma	13	0,016
Cell cycle	28	0,017
Adipocytokine signaling pathway	7	0,017
Proximal tubule bicarbonate reclamation	1	0,017
Chronic myeloid leukemia	16	0,017
Glutathione metabolism	3	0,018
Notch signaling pathway	5	0,018
alpha-Linolenic acid metabolism	2	0,019
Terpenoid backbone biosynthesis	2	0,019
Epithelial cell signaling in Helicobacter pylori infection	5	0,019
Toxoplasmosis	13	0,019
Epstein-Barr virus infection	14	0,019
Purine metabolism	16	0,02
Fat digestion and absorption	1	0,02
Glioma	14	0,02
Acute myeloid leukemia	9	0,02
Glycerolipid metabolism	5	0,023
PPAR signaling pathway	7	0,026
Pancreatic cancer	13	0,026
beta-Alanine metabolism	2	0,027
Riboflavin metabolism	1	0,028
Folate biosynthesis	1	0,028
Malaria	1	0,029
Tuberculosis	14	0,029
Pathways in cancer	42	0,029
Autoimmune thyroid disease	1	0,029
Fatty acid biosynthesis	1	0,03
Bile secretion	2	0,03
Glycosphingolipid biosynthesis - ganglio series	1	0,031
Ribosome biogenesis in eukaryotes	5	0,031
Shigellosis	6	0,031
Pyruvate metabolism	4	0,032
Transcriptional misregulation in cancer	2	0,032
Osteoclast differentiation	12	0,033
Chagas disease (American trypanosomiasis)	10	0,033
Measles	12	0,033
Phenylalanine metabolism	1	0,036
Salmonella infection	8	0,036
Oocyte meiosis	12	0,037
Pyrimidine metabolism	11	0,038
Vibrio cholerae infection	3	0,039
Antigen processing and presentation	3	0,041
Endocytosis	11	0,042
TGF-beta signaling pathway	11	0,042
VEGF signaling pathway	7	0,042
Arginine and proline metabolism	4	0,045
Amoebiasis	3	0,045
Influenza A	11	0,045
Systemic lupus erythematosus	1	0,047
Selenocompound metabolism	1	0,048
Chemokine signaling pathway	15	0,048
Circadian rhythm - mammal	3	0,048
Cell adhesion molecules (CAMs)	7	0,049

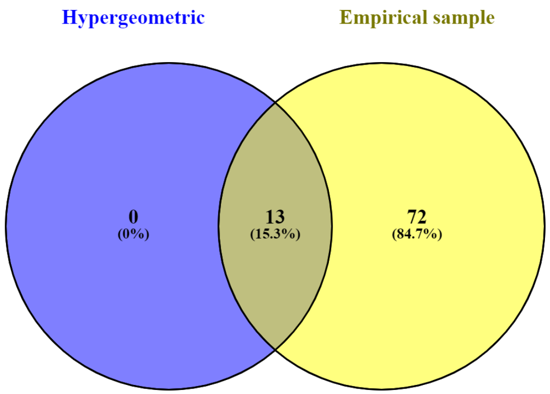

Supplement Table 7a

p53 signalling pathway	hsa-miR-223-3p	hsa-miR-24-3p	hsa-miR-486-5p	hsa-miR-941	total
APAF1	0	1	0	0	1
CCND1	0	1	0	0	1
CCNB1	0	1	0	0	1
CCNG1	0	1	0	0	1
CDK1	0	1	0	0	1
CDK4	0	1	1	0	2
CDKN2A	0	1	0	0	1
CHEK1	0	1	0	0	1
IGF1	0	1	0	0	1
MDM4	0	1	0	0	1
RRM2	0	1	0	0	1
TP53	1	1	0	0	2
PPM1D	0	1	0	0	1
BBC3	0	1	0	0	1
SESN1	0	1	0	0	1
STEAP3	0	1	0	0	1
RFWD2	0	1	0	0	1
ATM	1	0	0	0	1
CDK2	1	0	0	0	1
MDM2	1	0	0	0	1
SESN3	1	0	0	0	1
SERPINE1	0	0	1	0	1
PTEN	0	0	1	0	1
**Sum**	**5**	**17**	**3**	**0**	

Supplement Table 7b

Cell cycle	hsa-miR-223-3p	hsa-miR-24-3p	hsa-miR-486-5p	hsa-miR-941	total
CCND1	0	1	0	0	1
CCNA2	0	1	0	0	1
CCNB1	0	1	0	0	1
CDK1	0	1	0	0	1
CDK4	0	1	1	0	2
CDKN1B	0	1	0	0	1
CDKN2A	0	1	0	0	1
CHEK1	0	1	0	0	1
E2F1	1	1	0	0	2
E2F2	0	1	0	0	1
E2F3	0	1	0	0	1
HDAC1	0	1	0	0	1
MCM4	0	1	0	0	1
MYC	0	1	0	0	1
PCNA	0	1	0	0	1
RBL1	0	1	0	0	1
TFDP2	0	1	0	0	1
TGFB1	0	1	0	0	1
TP53	1	1	0	0	2
YWHAZ	0	1	0	0	1
PKMYT1	0	1	0	0	1
DBF4	0	1	0	0	1
ATM	1	0	0	0	1
CDC27	1	0	0	0	1
CDK2	1	0	0	0	1
MDM2	1	0	0	0	1
SMAD2	0	0	1	0	1
CDC20	0	0	0	1	1
**Sum**	**6**	**22**	**2**	**1**	
