# Supplementary material for: Next-generation sequencing analysis of circulating micro-RNA expression in response to parabolic flight as a spaceflight analogue
Source: NPJ Microgravity. 2020 Nov 2;6:31. doi: 10.1038/s41526-020-00121-9 (PMC7606465; doi:10.1038/s41526-020-00121-9)
Supplement: Supplementary file 2 — Reporting Summary [file 41526_2020_121_MOESM2_ESM.pdf]

nature research

Corresponding author(s): Christian Jung

Last updated by author(s): Sep 5, 2020

# Reporting Summary

Nature Research wishes to improve the reproducibility of the work that we publish. This form provides structure for consistency and transparency in reporting. For further information on Nature Research policies, see Authors & Referees and the Editorial Policy Checklist.

## Statistics

For all statistical analyses, confirm that the following items are present in the figure legend, table legend, main text, or Methods section.

| n/a | Confirmed | |
|---|---|---|
| ☐ | ☒ | The exact sample size (*n*) for each experimental group/condition, given as a discrete number and unit of measurement |
| ☐ | ☒ | A statement on whether measurements were taken from distinct samples or whether the same sample was measured repeatedly |
| ☐ | ☒ | The statistical test(s) used AND whether they are one- or two-sided<br>*Only common tests should be described solely by name; describe more complex techniques in the Methods section.* |
| ☐ | ☒ | A description of all covariates tested |
| ☐ | ☒ | A description of any assumptions or corrections, such as tests of normality and adjustment for multiple comparisons |
| ☐ | ☒ | A full description of the statistical parameters including central tendency (e.g. means) or other basic estimates (e.g. regression coefficient) AND variation (e.g. standard deviation) or associated estimates of uncertainty (e.g. confidence intervals) |
| ☒ | ☐ | For null hypothesis testing, the test statistic (e.g. *F*, *t*, *r*) with confidence intervals, effect sizes, degrees of freedom and *P* value noted<br>*Give P values as exact values whenever suitable.* |
| ☒ | ☐ | For Bayesian analysis, information on the choice of priors and Markov chain Monte Carlo settings |
| ☒ | ☐ | For hierarchical and complex designs, identification of the appropriate level for tests and full reporting of outcomes |
| ☒ | ☐ | Estimates of effect sizes (e.g. Cohen's *d*, Pearson's *r*), indicating how they were calculated |

*Our web collection on statistics for biologists contains articles on many of the points above.*

## Software and code

Policy information about availability of computer code

| | |
|---|---|
| Data collection | Data was collected and documented in Microsoft Excel |
| Data analysis | Statistical analysis of preprocessed NGS data was done with R v3.6 and the packages pheatmap v1.0.12, pcaMethods v1.78 and genefilter v1.68. Differential expression analysis with edgeR v3.28 (34). Regarding baseline characteristics and laboratory parameters, analysis was conducted using SPSS 25 (IBM SPSS Statistics, USA). |

For manuscripts utilizing custom algorithms or software that are central to the research but not yet described in published literature, software must be made available to editors/reviewers. We strongly encourage code deposition in a community repository (e.g. GitHub). See the Nature Research guidelines for submitting code & software for further information.

## Data

Policy information about availability of data

All manuscripts must include a data availability statement. This statement should provide the following information, where applicable:

- Accession codes, unique identifiers, or web links for publicly available datasets
- A list of figures that have associated raw data
- A description of any restrictions on data availability

Raw and normalized data were uploaded to NCBI Gene Expression Omnibus (32) and are accessible through GEO Series accession number GSE147380 (https://www.ncbi.nlm.nih.gov/geo/query/acc.cgi?acc=GSE147380).
Code: ehgjcyckhtgfxal

# Field-specific reporting

Please select the one below that is the best fit for your research. If you are not sure, read the appropriate sections before making your selection.

☒ Life sciences ☐ Behavioural & social sciences ☐ Ecological, evolutionary & environmental sciences

For a reference copy of the document with all sections, see nature.com/documents/nr-reporting-summary-flat.pdf

# Life sciences study design

All studies must disclose on these points even when the disclosure is negative.

| | |
|---|---|
| Sample size | No sample-size calculation was performed due to the hypothesis generating design of the study. Due to the limited availability of parabolic flight campaigns, we aimed at including as much participants as possible in the parabolic flight campaign. We were able to achieve a cohort of 8 individuals, all undergoing one flight day per subject. |
| Data exclusions | No data was excluded in our study. |
| Replication | Given the limited availability of parabolic flight campaigns, we could not replicate a participation in a campaign for this analyses. micro-RNA analyses used in our study are standardized and replicable. |
| Randomization | Due to the hypothesis generating design and the fact, that all study subjects underwent parabolic flight to acquire a high sample size, no randomization was conducted. Only healthy participants were included. |
| Blinding | Due to our study design (hypothesis generating - parabolic flight), blinding was not required and furthermore not possible. |

# Reporting for specific materials, systems and methods

We require information from authors about some types of materials, experimental systems and methods used in many studies. Here, indicate whether each material, system or method listed is relevant to your study. If you are not sure if a list item applies to your research, read the appropriate section before selecting a response.

## Materials & experimental systems

| n/a | Involved in the study | |
|---|---|---|
| ☒ | ☐ | Antibodies |
| ☒ | ☐ | Eukaryotic cell lines |
| ☒ | ☐ | Palaeontology |
| ☒ | ☐ | Animals and other organisms |
| ☐ | ☒ | Human research participants |
| ☒ | ☐ | Clinical data |

## Methods

| n/a | Involved in the study | |
|---|---|---|
| ☒ | ☐ | ChIP-seq |
| ☒ | ☐ | Flow cytometry |
| ☒ | ☐ | MRI-based neuroimaging |

## Human research participants

Policy information about studies involving human research participants

| | |
|---|---|
| Population characteristics | In total, 8 study participants were included in the study, 4 male and 4 females. The median age was 25.4 years. All participants were healthy without any comorbidities. |
| Recruitment | All participants were included in our Univerity clinic. As all participants had to be healthy without any comorbidities, our study collective was relatively young. However, given the healthy and young study sample, a selection bias influencing our findings can be excluded. |
| Ethics oversight | German Ethics Committee (Medical Faculty of the University Hospital Duesseldorf, Germany)<br>French Ethics Committee (Medical Faculty of the University of Caen). |

Note that full information on the approval of the study protocol must also be provided in the manuscript.
